# Wnt-β-Catenin Signaling in Human Dendritic Cells Mediates Regulatory T-Cell Responses to Fungi via the PD-L1 Pathway

**DOI:** 10.1128/mBio.02824-21

**Published:** 2021-11-16

**Authors:** Anupama Karnam, Srinivasa Reddy Bonam, Naresh Rambabu, Sarah Sze Wah Wong, Vishukumar Aimanianda, Jagadeesh Bayry

**Affiliations:** a Institut National de la Santé et de la Recherche Médicale, Centre de Recherché des Cordeliers, Sorbonne Université, Université de Paris, Paris, France; b Institut Pasteur, Molecular Mycology Unit, CNRS UMR2000, Paris, France; c Department of Biological Sciences & Engineering, Indian Institute of Technology Palakkad, Palakkad, India; Universidade de Sao Paulo

**Keywords:** *Aspergillus fumigatus*, regulatory T cells, dendritic cells, Wnt-β-catenin, PD-L1, DC-SIGN, dectin-1, dectin-2, human, Wnt signaling

## Abstract

The signaling pathways activated following interaction between dendritic cells (DCs) and a pathogen determine the polarization of effector T-cell and regulatory T-cell (Treg) responses to the infection. Several recent studies, mostly in the context of bacterial infections, have shown that the Wnt/β-catenin pathway plays a major role in imparting tolerogenic features in DCs and in promotion of Treg responses. However, the significance of the Wnt/β-catenin pathway’s involvement in regulating the immune response to the fungal species is not known. Using Aspergillus fumigatus, a ubiquitous airborne opportunistic fungal species, we show here that fungi activate the Wnt/β-catenin pathway in human DCs and are critical for mediating the immunosuppressive Treg responses. Pharmacological inhibition of this pathway in DCs led to inhibition of maturation-associated molecules and interleukin 10 (IL-10) secretion without affecting the majority of the inflammatory cytokines. Furthermore, blockade of Wnt signaling in DCs suppressed DC-mediated Treg responses in CD4^+^ T cells and downregulated both tumor necrosis factor alpha (TNF-α) and IL-10 responses in CD8^+^ T cells. Mechanistically, induction of β-catenin pathway by A. fumigatus required C-type lectin receptors and promoted Treg polarization via the induction of programmed death-ligand 1 on DCs. Further investigation on the identity of fungal molecular patterns has revealed that the cell wall polysaccharides β-(1, 3)-glucan and α-(1, 3)-glucan, but not chitin, possess the capacity to activate the β-catenin pathway. Our data suggest that the Wnt/β-catenin pathway is a potential therapeutic target to selectively suppress the Treg response and to sustain the protective Th1 response in the context of invasive aspergillosis caused by A. fumigatus.

## INTRODUCTION

Aspergillus fumigatus is an omnipresent airborne fungal pathogen. Human lungs are constantly exposed to A. fumigatus conidia (asexual spores, and are airborne) through breathing. Although inhaled conidia are usually eliminated in healthy individuals, they may cause hypersensitization, severe asthma with fungal sensitization, allergic bronchopulmonary aspergillosis, colonization of altered respiratory epithelium, and aspergilloma in existing pulmonary lesions; they can lead to life-threatening invasive aspergillosis in immunocompromised hosts (1–5).

Innate immune cells, including macrophages, dendritic cells (DCs), and neutrophils, are involved in antifungal activity against A. fumigatus. Studies have shown that macrophages are involved in A. fumigatus conidial clearance, whereas germinating morphotypes of A. fumigatus are destroyed by neutrophils through the release of iron chelator (as iron is essential for germination) and reactive oxygen species (ROS) and by forming neutrophil extracellular traps. DCs can also internalize both conidial and hyphal morphotypes of A. fumigatus and could undergo functional maturation (6). Being professional antigen-presenting cells, DCs are also involved in polarizing distinct CD4^+^ T-cell responses (7–10). Upon fungal encounter, DCs engage their various pattern recognition receptors (PRRs) to recognize the evading pathogen (11–13). These A. fumigatus-educated DCs subsequently instruct distinct CD4^+^ T-cell polarization like Th1, Th2, Th17, and FoxP3^+^ regulatory T cells (Tregs). Among these, Th2 and Th17 responses are nonprotective for Aspergillus infection. On the other hand, Th1 cells have a major role for the induction of protective immune responses (14–17). Although Tregs are immunosuppressive and promote chronic and persistent infection, they are also critical for preventing inflammation-associated tissue damage (18, 19). Therefore, the balance between Th1 and Treg responses is critical for the protective immune response against A. fumigatus.

During pathogen-DC interaction, the signaling pathways activated and cytokines secreted by DCs play a vital role in deciding T-cell responses to the infection. In this regard, recent studies have demonstrated the involvement of Wnt/β-catenin pathway for the induction of tolerogenic functions in DCs and promotion of Treg responses via various anti-inflammatory mechanisms, like expression of IL-10, transforming growth factor beta (TGF-β), and retinoic acid (20–24). Wnt signaling is an evolutionarily preserved pathway present in all cell types. β-Catenin is the principal molecule of the canonical Wnt pathway and during the “Wnt off” state, it is constantly phosphorylated by casein kinase-1 (CK-1) (at Ser45) and glycogen synthase kinase-3 beta (GSK-3β) (at Thr41, Ser37, and Ser33). The phosphorylated β-catenin is recognized and tagged by ubiquitin molecules for proteasomal degradation. Binding of Wnt ligands to the Frizzled receptor activates the Wnt/β-catenin pathway and inactivates the negative regulator GSK-3β by phosphorylation (p-GSK-3β). As a consequence, the nonphosphorylated active form of β-catenin accumulates in the cytoplasm and subsequently translocates to the nucleus, where it acts as a transcription cofactor to control the expression of various genes by interacting with T-cell factor/lymphoid enhancer-binding factor (TCF/LEF) (25, 26).

Several reports have highlighted the role of the Wnt/β-catenin pathway in mediating the immune surveillance functions of innate immune cells against invading pathogens and in particular against bacteria (27–30). However, the implication of this pathway in the regulation of immune response to fungal species is not well studied. Earlier report has demonstrated that inhibition of GSK-3β in immature DCs with lithium chloride (LiCl) or SB415286, as well as transfection with small interfering RNA, leads to markedly elevated expression of the anti-inflammatory cytokine IL-10 (31). A. fumigatus infection of human/mouse cornea or human monocyte cell line THP-1 induced the production of Wnt5a, the Wnt ligand, in a dectin-1- and LOX-1-dependent manner (32). Infection of mouse peritoneal macrophages with different fungal pathogens or with curdlan [linear β-(1, 3)-glucan produced by Alcaligenes faecalis] also led to dectin-1-dependent WNT5A expression (33).

By using A. fumigatus as a model, here we show that fungal species activate the Wnt/β-catenin pathway in human DCs, along with the secretion of Wnt ligands Wnt1 and Wnt7a. Inhibition of the Wnt pathway prior to stimulation with A. fumigatus resulted in decreased DC maturation and selective inhibition of anti-inflammatory cytokine IL-10 without affecting the secretion of most of the proinflammatory cytokines. Abrogation of the Wnt/β-catenin pathway in DCs also led to reduced Treg polarization without altering the polarization of other CD4^+^ T-cell subsets. Mechanistically, induction of the β-catenin pathway by A. fumigatus in DCs required C-type lectin receptors and mediated Treg responses via induction of programmed death ligand 1 (PD-L1) on DCs. Dissection of the identity of A. fumigatus pathogen-associated molecular patterns (PAMPs) revealed that the cell wall polysaccharides β-(1, 3)-glucan and α-(1, 3)-glucan, but not chitin, possess the capacity to activate the β-catenin pathway in human DCs.

## RESULTS

### A. fumigatus swollen conidia, live conidia, and hyphae, but not dormant conidia, activate the Wnt/β-catenin pathway in human DCs.

**FIG 1:**
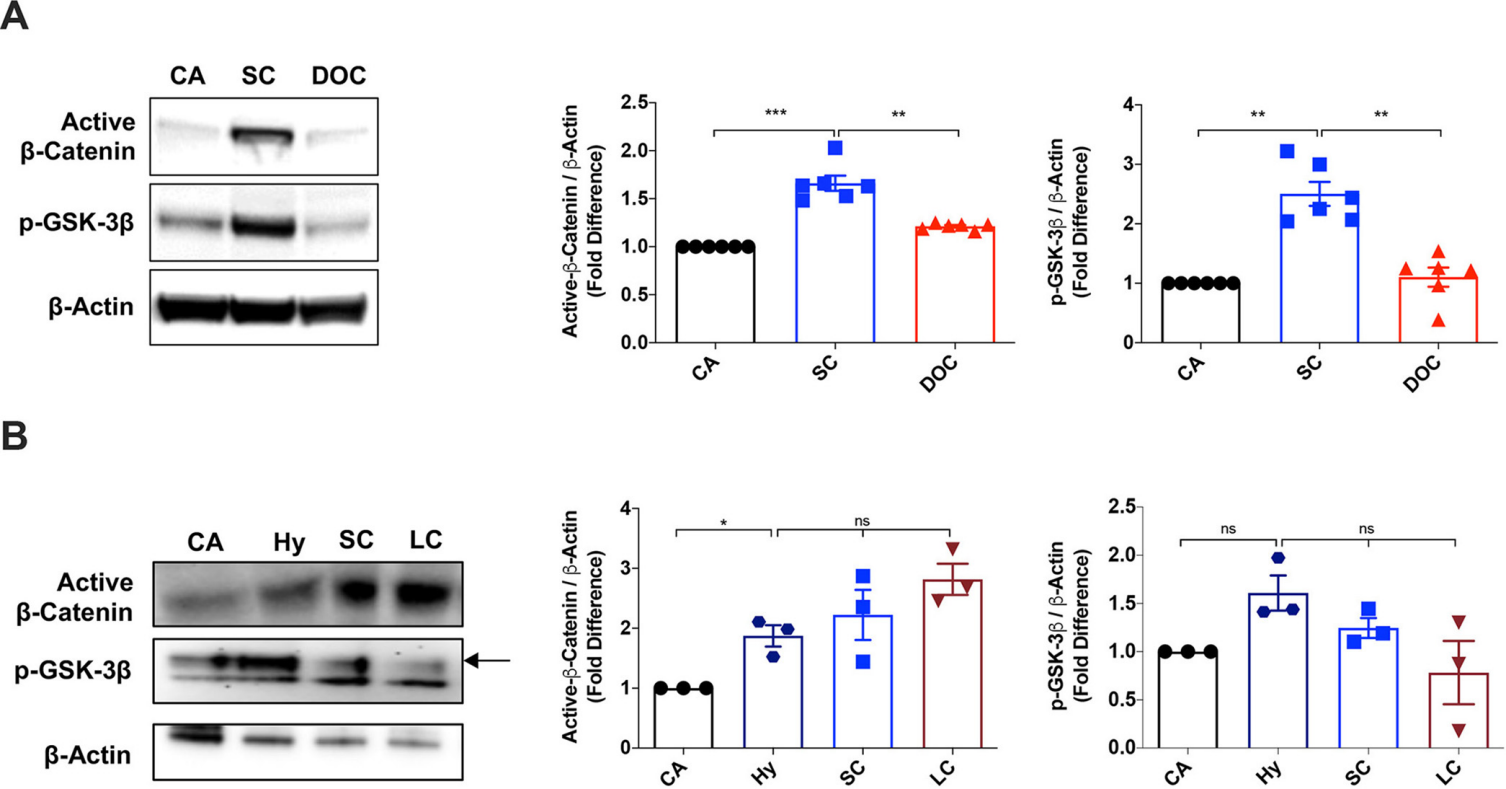
A. fumigatus swollen conidia and live conidia, as well as hyphae, activate the Wnt/β-catenin pathway in human (DCs). (A) Monocyte-derived DCs (0.5 × 10^6^ cells/mL) were cultured with granulocyte-macrophage colony-stimulating factor (GM-CSF) and interleukin 4 (IL-4) and were either unstimulated (CA) or stimulated with swollen conidia (SC) (0.5 × 10^6^/mL) or dormant conidia (DOC) (0.5 × 10^6^/mL) for 24 h. The activation of Wnt/β-catenin pathway was assessed by immunoblotting of active β-catenin and p-GSK-3β proteins. β-Actin was used as a protein loading control. Representative blot and densitometric analyses of the blots for active β-catenin and p-GSK-3β proteins (mean ± standard error of the mean [SEM]; *n* = 6 independent donors) are presented. (B) Monocyte-derived DCs (0.5 × 10^6^ cells/mL) were cultured with GM-CSF and IL-4 and were either unstimulated (CA) or stimulated with germinating/hyphae morphotype (Hy; 0.5 × 10^6^/mL), SC (0.5 × 10^6^/mL), or live conidia (LC; 500/mL) for 24 h. Representative blot and densitometric analyses of the blots for active β-catenin and p-GSK-3β proteins (mean ± SEM; *n* = 3 independent donors) are presented. *, *P* < 0.05; **, *P* < 0.01; ***, *P* < 0.001; ns, not significant; one-way analysis of variance (ANOVA) with Dunnett’s multiple-comparison test.

To understand the role of the Wnt/β-catenin pathway during A. fumigatus infection, we first assessed the status of this pathway by immunoblot-based evaluation of the levels of active β-catenin and p-GSK-3β in DCs upon stimulation with swollen conidia. We used swollen conidia in our study, as dormant conidia that are covered by the rodlet/melanin layer are immunologically inert (34), while swollen morphotypes of conidia expose PAMPs on their surfaces (35). As the A. fumigatus cell wall is a dynamic structure undergoing modification during germination, we used paraformaldehyde-inactivated conidia to avoid any interference due to the metabolic activity of the fungus. Human monocyte-derived DCs stimulated with paraformaldehyde-inactivated A. fumigatus swollen conidia (here onwards termed swollen conidia) for 24 h showed increased levels of active β-catenin and p-GSK-3β, indicating the activation of the Wnt/β-catenin pathway (Fig. 1A). On the other hand, dormant conidia did not activate the pathway. Indeed, the levels of active β-catenin and p-GSK-3β in the dormant conidia-treated condition were similar to those of untreated DCs (Fig. 1A). The ability to induce the Wnt/β-catenin pathway was not restricted to swollen conidia. A germinating/hyphal morphotype, as well as metabolically active live A. fumigatus conidia, could also induce activation of this pathway, thus validating the physiological relevance of our observations (Fig. 1B).

### A. fumigatus swollen conidia induce secretion of Wnt1 and Wnt7a ligands.

**FIG 2:**
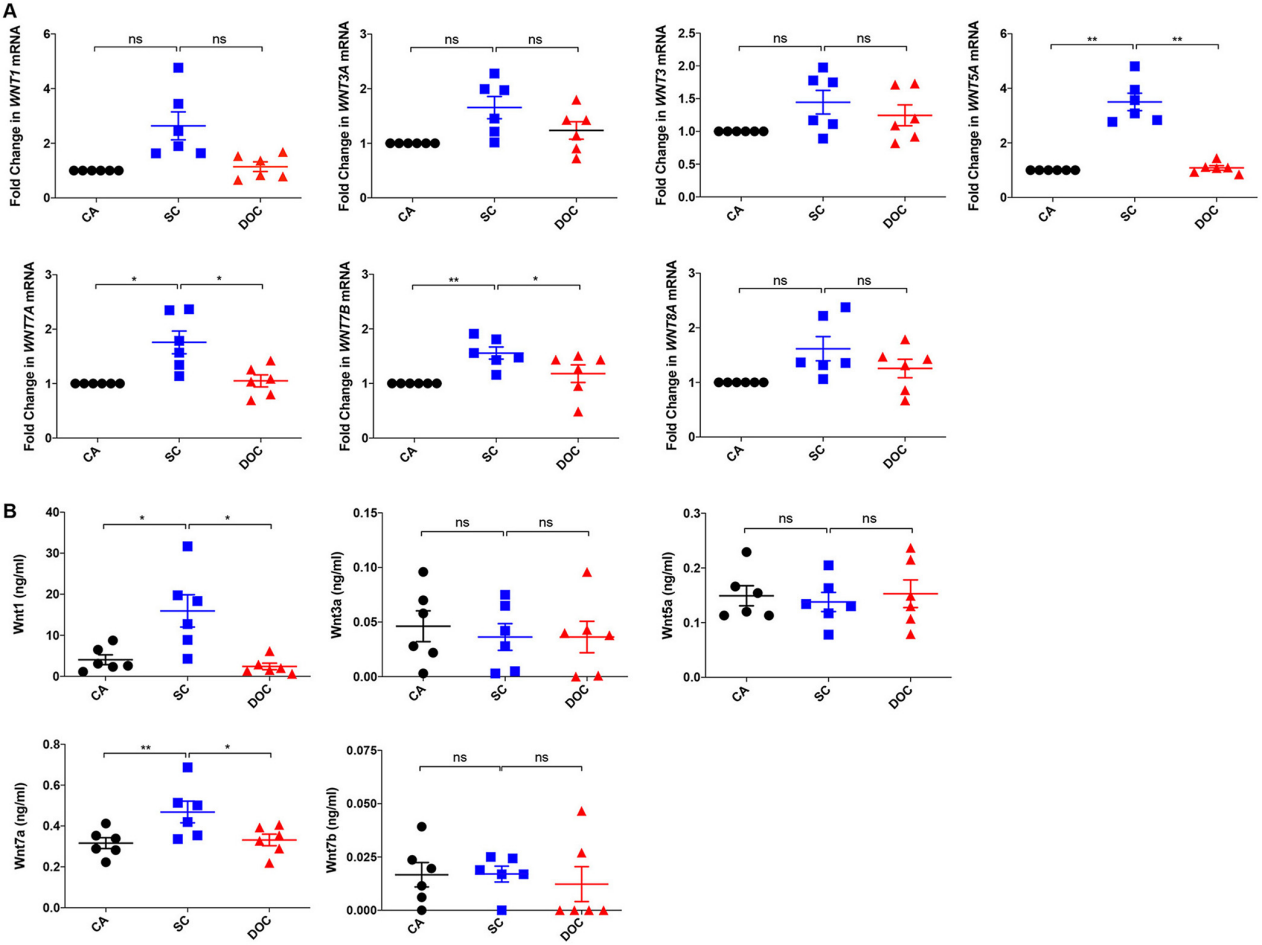
A. fumigatus swollen conidia induce secretion of Wnt1 and Wnt7a proteins in DCs. DCs (0.5 × 10^6^ cells/mL) were cultured with GM-CSF and IL-4 and were either stimulated with swollen conidia (SC) or dormant conidia (DOC), for 18 h, or kept unstimulated as a control (CA). (A) Expression of various canonical (*WNT1*, *WNT3A*, *WNT3*, and *WNT8A*) and noncanonical Wnt ligands (*WNT5A*, *WNT7A*, and *WNT7B*) (mean ± SEM; *n* = 6 donors) was analyzed by quantitative real-time reverse transcription-PCR (RT-PCR); fold change in the expression of these genes compared to that of the housekeeping gene GAPDH is plotted. (B) The amount (ng/mL) of Wnt1, Wnt3a, Wnt5a, Wnt7a, and Wnt7b in the cell-free supernatants (mean ± SEM; *n* = 6 donors) after 24 h of stimulation of DCs with swollen conidia or dormant conidia. *, *P* < 0.05; **, *P* < 0.01; ns, not significant; determined by one-way ANOVA followed by Tukey’s multiple-comparison test.

Typically, binding of Wnt ligands to the Frizzled receptor initiates a series of events leading to inactivation of GSK-3β, followed by translocation of active β-catenin to the nucleus. Wnt ligands carry out their function in both an autocrine and a paracrine manner. Humans express 19 Wnt genes; however, not all of them are involved in the activation of the Wnt/β-catenin pathway (36). By quantitative reverse transcription-PCR (RT-PCR), we screened for Wnt genes in DCs, including *WNT1*, *WNT3*, *WNT3A*, *WNT5A*, *WNT7A*, *WNT7B*, and *WNT8A* that are capable of activating β-catenin signaling. We found that the levels of *WNT5A*, *WNT7A*, and *WNT7B* were significantly higher in A. fumigatus swollen conidia-stimulated DCs than those in unstimulated cells and dormant conidia-stimulated DCs (Fig. 2A).

Furthermore, to confirm these results, we analyzed the amounts of Wnt5a, Wnt7a, Wnt7b, and typical canonical Wnt ligands like Wnt1 and Wnt3a secreted into the cell culture supernatants. Although genes encoding all of these proteins were upregulated upon stimulation with swollen conidia, we observed the secretion of only Wnt1 and Wnt7a, but not Wnt3a, Wnt5a, and Wnt7b, in the supernatants of DCs stimulated with swollen conidia (Fig. 2B). Together, these data demonstrate that A. fumigatus swollen conidia activate the Wnt/β-catenin pathway with the concomitant secretion of Wnt1 and Wnt7a ligands by human DCs.

### Wnt/β-catenin signaling plays a critical role in A. fumigatus swollen conidia-induced maturation of human DCs.

**FIG 3:**
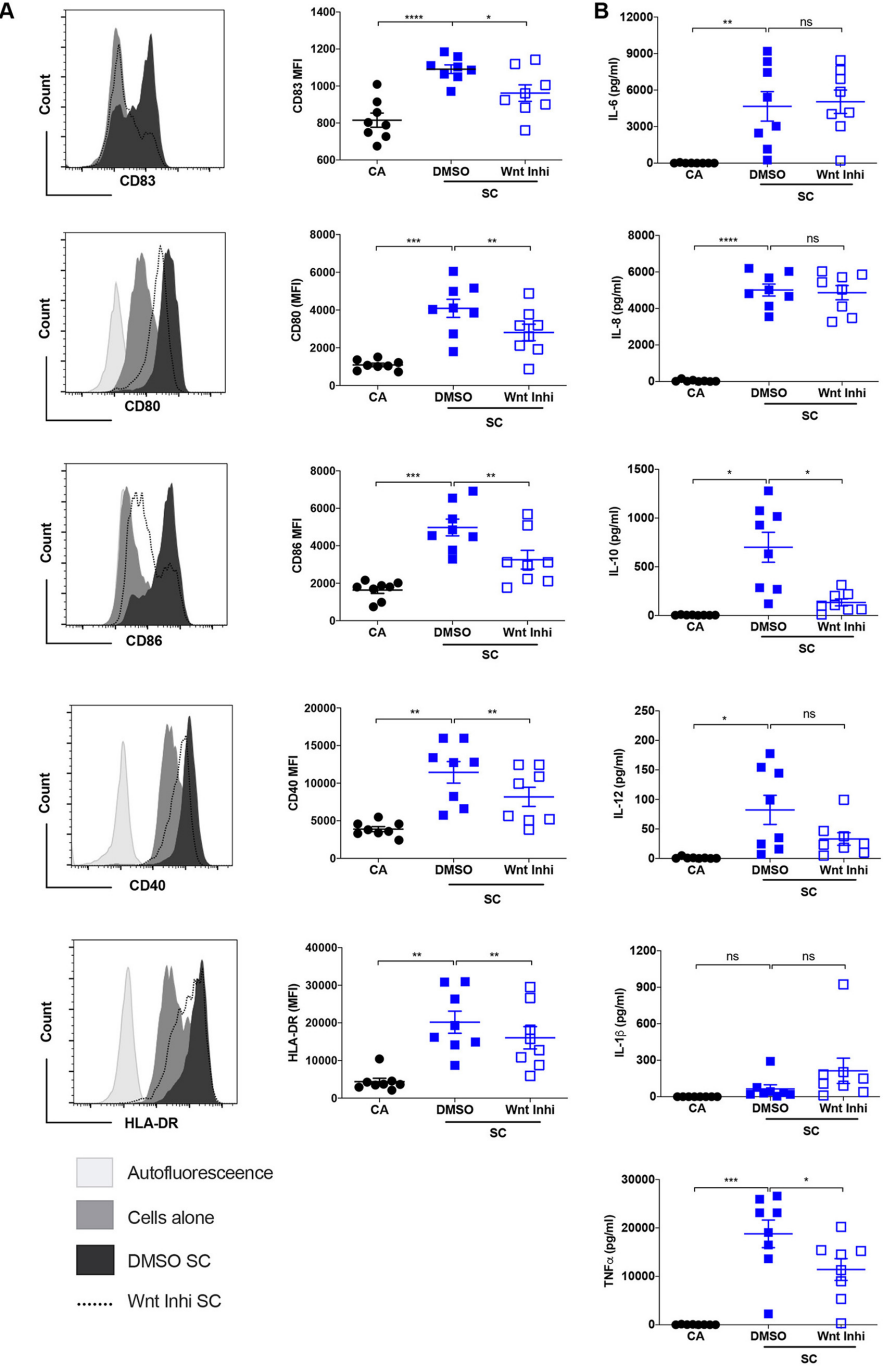
Wnt/β-catenin signaling is critical for A. fumigatus swollen conidia to induce maturation of DCs. DCs (0.5 × 10^6^ cells/mL) were cultured with GM-CSF and IL-4 and were either unstimulated (CA) or were treated with dimethyl sulfoxide (DMSO) or Wnt inhibitor (Wnt Inhi) for 2 h followed by stimulation with swollen conidia (SC) at a 1:1 ratio for 48 h. DC phenotype was analyzed by flow cytometry. (A) Representative histogram overlays showing the expression of CD83, CD80, CD86, CD40, and HLA-DR on DCs under various experimental conditions and median fluorescence intensities (MFI) (mean ± SEM; *n* = 8 donors) of those markers. (B) The amount (pg/mL) of secreted interleukin 6 (IL-6), IL-8, IL-10, IL-12, IL-1β, and tumor necrosis factor alpha (TNF-α) cytokines in the cell-free supernatant from the above-described experiments (mean ± SEM; *n* = 8 donors) are measured by an enzyme-linked immunosorbent assay (ELISA). *, *P* < 0.05; **, *P* < 0.01; ***, *P* < 0.001; ****, *P* < 0.0001; ns, not significant as determined by one-way ANOVA test with Tukey’s multiple-comparison test.

We then explored the importance of Wnt signaling in the crosstalk between DCs and A. fumigatus by employing the pharmacological inhibitor C-59. Wnt inhibition reduced the phosphorylation of GSK-3β (see Fig. S1 in the supplemental material). Blockade of Wnt signaling in DCs subdued the ability of A. fumigatus swollen conidia to induce the maturation of DCs. The expression of CD83; costimulatory molecules like CD80, CD86, CD40; and the antigen-presenting molecule HLA-DR was significantly downregulated upon inhibition of Wnt signaling compared that in swollen conidia-stimulated DCs (Fig. 3A). Similar results were also obtained with live conidia, where the blockade of Wnt signaling in DCs before infection with live conidia inhibited the expression of various DC maturation-associated molecules (Fig. S2).

10.1128/mBio.02824-21.1 FIG S1 Effect of Wnt inhibition on the phosphorylation of GSK-3β in dendritic cells (DCs). DCs (0.5 × 10^6^ cells/mL) were treated with dimethyl sulfoxide (DMSO) or Wnt inhibitor (Wnt Inhi) for 2 h, followed by stimulation with swollen conidia (SC) at a 1:1 ratio for 24 h. Immunoblotting was performed for the analyses of p-GSK-3β. β-Actin was used as a protein loading control. Representative blot and densitometric analyses of the blots for p-GSK-3β protein (mean ± standard error of the mean [SEM]; *n* = 4 independent donors) are presented. *P* = 0.06; one-way analysis of variance (ANOVA) with Dunnett’s multiple-comparisons test. Download FIG S1, EPS file, 0.3 MB. Copyright © 2021 Karnam et al. 2021 Karnam et al. https://creativecommons.org/licenses/by/4.0/ This content is distributed under the terms of the Creative Commons Attribution 4.0 International license.

10.1128/mBio.02824-21.2 FIG S2 Inhibition of Wnt/β-catenin signaling reduces the expression of live Aspergillus fumigatus conidia-induced maturation markers on human DCs. DCs (0.5 × 10^6^ cells/mL) were cultured with GM-CSF and interleukin 4 (IL-4) and were either unstimulated (CA) or treated with DMSO or Wnt inhibitor (Wnt Inhi) for 2 h, followed by stimulation with live conidia (LC; *n* = 500) for 48 h. The expression of CD80, CD86, CD274, CD40, and HLA-DR on DCs was analyzed by flow cytometry. Data (mean ± SEM) are from 4 donors. *, *P* < 0.05; **, P < 0.01; ***, P < 0.001; ns, not significant as determined by one-way ANOVA test with Tukey’s multiple-comparisons test. Download FIG S2, EPS file, 0.5 MB. Copyright © 2021 Karnam et al. 2021 Karnam et al. https://creativecommons.org/licenses/by/4.0/ This content is distributed under the terms of the Creative Commons Attribution 4.0 International license.

Cytokines secreted by DCs play a decisive role in mounting T-cell responses. Although we observed a significant decrease in the ability of swollen conidia to induce DC maturation upon inhibition of Wnt signaling, the levels of proinflammatory cytokines like IL-6 and IL-8 remained similar to those under swollen conidia-stimulated conditions (Fig. 3B). In addition, a marginal but nonsignificant decline in the levels of IL-12 was observed (Fig. 3B). IL-1β was marginally induced by swollen conidia and was not altered upon inhibition of Wnt signaling (Fig. 3B). Interestingly, the levels of TNF-α and the anti-inflammatory cytokine IL-10 were downregulated (Fig. 3B) in the DCs treated with Wnt inhibitor, pointing toward potential anti-inflammatory nature of the Wnt signaling in response to A. fumigatus. In line with these data, a previous report demonstrated that inhibition of GSK-3 in DCs before infection with A. fumigatus germlings led to an enhanced expression of IL-10 (31).

### Active Wnt signaling in DCs is essential for the polarization of Treg response to A. fumigatus.

We then investigated the importance of Wnt/β-catenin signaling in DCs to induce different CD4^+^ T-cell polarization by A. fumigatus swollen conidia. To study this, DCs were exposed to Wnt inhibitor, followed by stimulation with swollen conidia. After 48 h, DCs were washed and cocultured with autologous naive CD4^+^ T cells for 5 days. Different subsets of CD4^+^ T cells were analyzed based on intracellular staining for cytokines IFN-γ, IL-17A, and IL-4 for Th1, Th17, and Th2 cells, respectively. FoxP3, along with the surface expression of CD25 and CD127, was used for the identification of Tregs.

**FIG 4:**
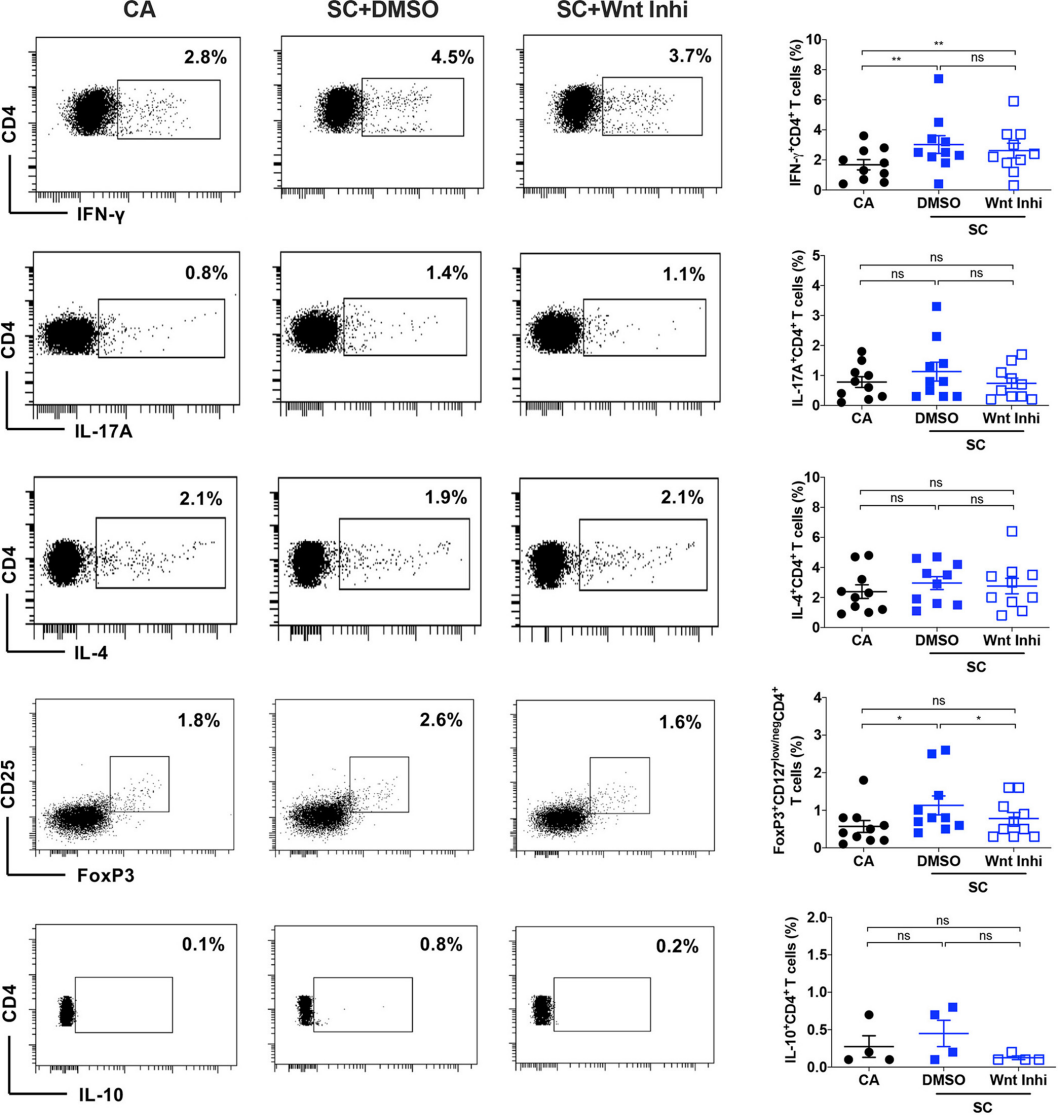
Wnt/β-catenin signaling is critical for A. fumigatus swollen conidia-stimulated DCs to induce polarization of Treg response. DCs (0.5 × 10^6^ cells/mL) were cultured with GM-CSF and IL-4 and were either unstimulated (CA) or exposed to DMSO or Wnt inhibitor (Wnt Inhi) for 2 h, followed by stimulation with swollen conidia (SC). After 48 h, DCs were washed and cocultured with autologous naive CD4^+^ T cells for 5 days. The polarization of various T-cell subsets was analyzed by flow cytometry. Representative dot plots (% positive cells) and pooled data (mean ± SEM) from 4 to 10 donors showing the frequency of Th1 cells (IFN-γ^+^ CD4^+^), Th17 cells (IL-17A^+^ CD4^+^), Th2 (IL-4^+^ CD4^+^) cells, Tregs (CD25^+^ FoxP3^+^ CD127^low/neg^ CD4^+^), and IL-10-secreting CD4^+^ T cells (IL-10^+^ CD4^+^) were presented. *, *P* < 0.05; **, *P* < 0.01; ns, not significant as determined by one-way ANOVA with Tukey’s multiple-comparison test.

Swollen conidia-stimulated DCs polarized mainly Th1 and Treg responses, validating our previous results (Fig. 4) (37). In line with the observation that Wnt signaling had no impact on the secretion of majority of the inflammatory cytokines in DCs, the ability of swollen conidia-stimulated DCs to polarize Th1, Th17, or Th2 cells from naive T cells remained intact upon blockade of Wnt/β-catenin pathway in DCs (Fig. 4). On the other hand, the capacity of DCs to polarize Tregs was significantly declined upon inhibition of Wnt/β-catenin pathway (Fig. 4). These data together suggest that the Wnt/β-catenin pathway in DCs promotes polarization of CD4^+^ T cells into Treg cells upon A. fumigatus stimulation.

Using peripheral blood mononuclear cells (PBMCs) isolated from invasive aspergillosis patients, it was shown that stimulation of PBMCs with various A. fumigatus antigens leads to the expansion of IL-10-producing T cells (38). However, in our experimental setup, we did not observe IL-10 induction in expanded CD4^+^ T cells (Fig. 4). Further, inhibition of GSK-3β by LiCl in DCs did not impact either Treg polarization or IL-10 induction (Fig. S3).

10.1128/mBio.02824-21.3 FIG S3 Repercussion of GSK-3β inhibition towards CD4^+^ T-cell responses induced by A. fumigatus swollen conidia-stimulated DCs. DCs (0.5 × 10^6^ cells/mL) were cultured with granulocyte-macrophage colony-stimulating factor (GM-CSF) and IL-4 and were either unstimulated (CA) or exposed to LiCl inhibitor for 2 h, followed by stimulation with swollen conidia (SC). After 48 h, DCs were washed and cocultured with autologous naive CD4^+^ T cells for 5 days. Frequencies (mean ± SEM; *n* = 4) of Th1 cells (IFN-γ^+^ CD4^+^), Th17 cells (IL-17A^+^ CD4^+^), Tregs (CD25^+^ FoxP3^+^ CD127^low/neg^ CD4^+^) and IL-10-secreting CD4^+^ T cells (IL-10^+^ CD4^+^) are presented. ns, not significant as determined by one-way ANOVA with Tukey’s multiple-comparisons test. Download FIG S3, EPS file, 0.5 MB. Copyright © 2021 Karnam et al. 2021 Karnam et al. https://creativecommons.org/licenses/by/4.0/ This content is distributed under the terms of the Creative Commons Attribution 4.0 International license.

### Wnt/β-catenin signaling in DCs regulates CD8^+^ T-cell response to A. fumigatus.

**FIG 5:**
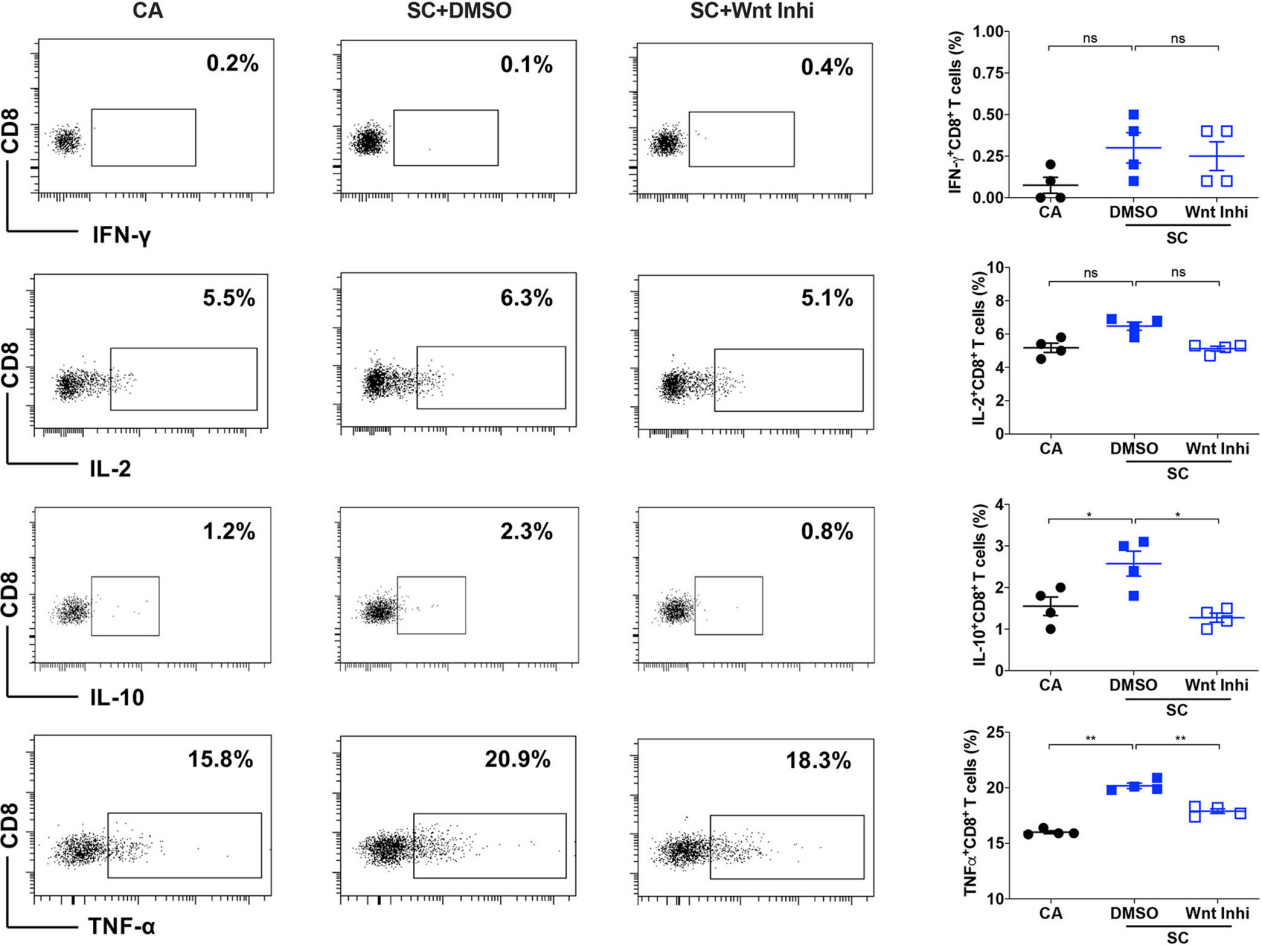
Wnt/β-catenin signaling in DCs regulate CD8^+^ T-cell response to A. fumigatus. DCs (0.5 × 10^6^ cells/mL) were cultured with GM-CSF and IL-4 and were either unstimulated (CA) or exposed to DMSO or Wnt inhibitor (Wnt Inhi) for 2 h followed by stimulation with swollen conidia (SC). After 48 h, DCs were washed and cocultured with autologous CD8^+^ T cells for 5 days. CD8^+^ T-cell response was analyzed based on the intracellular staining for the cytokines gamma interferon (IFN-γ), IL-2, IL-10, and TNF-α. Representative dot plots (% positive cells) and pooled data (mean ± SEM) from 4 donors were presented. *, *P* < 0.05; **, *P* < 0.01; ns, not significant as determined by one-way ANOVA with Tukey’s multiple-comparison test.

Next, we investigated the role of Wnt/β-catenin signaling in DCs to induce CD8^+^ T-cell responses to A. fumigatus swollen conidia. CD8^+^ T cells also play an important role in protection against A. fumigatus infection (6). DCs were treated with Wnt inhibitor and then stimulated with swollen conidia for 48 h. DCs were washed and cocultured with autologous CD8^+^ T cells for 5 days. CD8^+^ T-cell response was analyzed based on intracellular staining for the cytokines IFN-γ, IL-2, IL-10, and TNF-α. Swollen conidia-stimulated DCs induced mainly TNF-α and IL-10, while IFN-γ and IL-2 were not induced (Fig. 5). Moreover, inhibition of the Wnt/β-catenin pathway in DCs significantly downregulated both TNF-α and IL-10 responses in CD8^+^ T cells (Fig. 5).

### The Wnt/β-catenin pathway regulates the expression of PD-L1 on DCs.

We next aimed to identify the mechanism by which Wnt/β-catenin pathway regulates Treg response to A. fumigatus. Various costimulatory molecules on DCs play a vital role in regulating the polarization of Tregs from naive T cells (39). The interaction between PD-1, OX-40, and ICOS on CD4^+^ T cells and PD-L1 (CD274)/PD-L2 (CD273), OX-40L (CD252), and ICOSL (CD275) on DCs have been shown to induce Tregs under particular experimental conditions (37, 40–44). Hence, we examined the involvement of these costimulatory molecules in the Wnt pathway-mediated polarization of Tregs.

**FIG 6:**
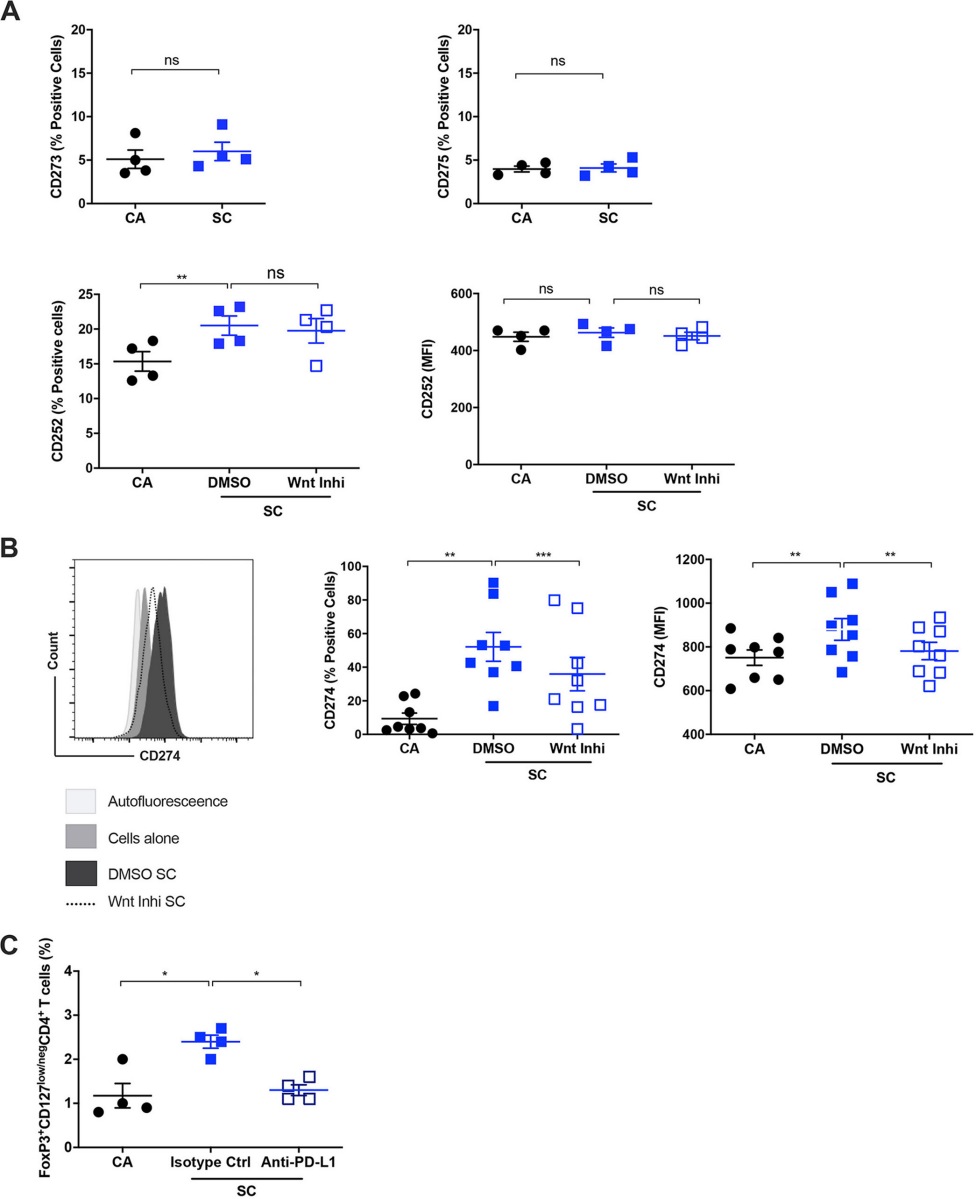
Wnt/β-catenin signaling regulates the A. fumigatus-induced expression of PD-L1 on DCs. DCs (0.5 × 10^6^ cells/mL) were cultured with GM-CSF and IL-4 and were either left unstimulated (CA) or treated with DMSO or Wnt inhibitor (Wnt Inhi) for 2 h followed by stimulation with swollen conidia (SC). After 48 h, the expressions of CD273 (PD-L2), CD275 (ICOSL), CD252 (OX-40L), and CD274 (PD-L1) were examined by flow cytometry. (A) Expression of CD273, CD275 (% positive cells), and CD252 (% positive cells and median fluorescence intensity [MFI]) on A. fumigatus swollen conidia-stimulated DCs (mean ± SEM; *n* = 2 in duplicates) with or without inhibition of Wnt signaling. (B) Expression of CD274 (PD-L1) on swollen conidia-stimulated DCs with or without inhibition of Wnt signaling. Representative histogram overlays, percent positive cells and MFI of CD274 (mean ± SEM; *n* = 8 independent donors) were indicated. (C) PD-L1 blockade abrogates the ability of swollen conidia-stimulated DCs to induce Treg expansion. DCs (0.5 × 10^6^ cells/mL) were cultured with GM-CSF and IL-4 and were either unstimulated (CA) or stimulated with swollen conidia (SC). After 48 h, cells were washed and stimulated DCs were incubated with either blocking monoclonal antibodies to PD-L1 or isotype control antibodies and then cocultured with autologous naive CD4^+^ T cells for 5 days. The frequency of Tregs was analyzed by flow cytometry. The data (mean ± SEM) from 4 donors were presented. *, *P* < 0.05; **, *P* < 0.01; ***, *P* < 0.001; ns, not significant as determined by one-way ANOVA followed by Tukey’s multiple-comparison test.

We found that swollen conidia did not induce the expression of PD-L2 and ICOSL on DCs and that the expression of these molecules remained at the basal level (Fig. 6A). On the other hand, OX-40L expression (percentage value) was marginally increased, but blockade of Wnt signaling in DCs did not affect the expression of this costimulatory molecule (Fig. 6A). It is of note that the expression of PD-L1 was increased significantly after stimulation with swollen conidia (Fig. 6B) and live conidia (Fig. S2). Furthermore, the ability of swollen conidia to induce the expression of this costimulatory molecule was significantly affected when Wnt pathway was inhibited (Fig. 6B).

These data suggested that Wnt/β-catenin signaling promotes Treg polarization by acting as an upstream regulator of the expression of PD-L1 in human DCs. In order to validate that enhanced PD-L1 expression on DCs is involved in the polarization of CD4^+^ T cells into Tregs, we blocked PD-L1 on DCs before coculture with naive CD4^+^ T cells. We found that PD-L1 blockade led to significant downregulation of Treg polarization (Fig. 6C).

### A. fumigatus cell wall glucans, but not chitin, activate the Wnt/β-catenin pathway in human DCs.

**FIG 7:**
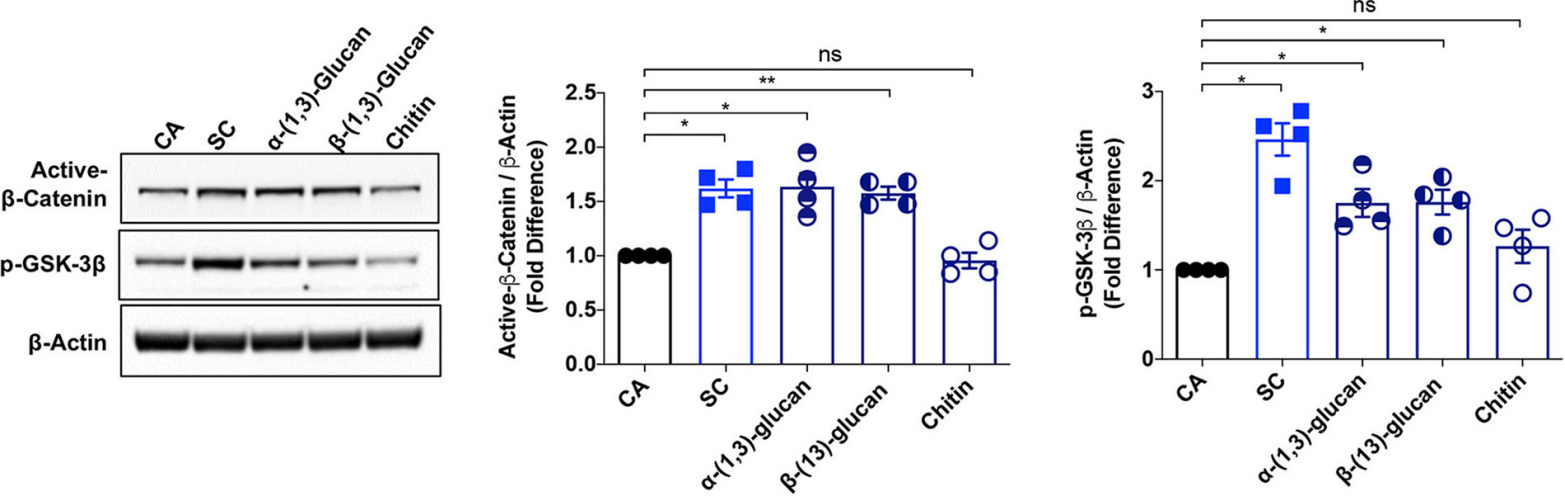
A. fumigatus cell wall glucans but not chitin activate Wnt/β-catenin signaling in DCs. DCs were cultured with GM-CSF and IL-4 and were either unstimulated (CA) or stimulated with swollen conidia (SC) (0.5 × 10^6^/mL), or with α-(1, 3)-glucan (1 μg/mL/0.5 × 10^6^ cells), or with β-(1, 3)-glucan (1 μg/mL/0.5 × 10^6^ cells), or with chitin (1 μg/mL/0.5 × 10^6^ cells) for 24 h. Activation of Wnt/β-catenin pathway was analyzed by immunoblotting of active β-catenin and p-GSK-3β. Actin was used as a loading control. Representative blots and densitometric analyses (mean ± SEM; *n* = 4) are displayed. *, *P* < 0.05; **, *P* < 0.01; ns, not significant; as determined by one-way ANOVA followed by Dunnett’s multiple-comparison test.

The cell wall of A. fumigatus is a complex structure composed of different polysaccharides, which act as PAMPs. The major PAMPs of A. fumigatus cell wall are β-(1,3)-glucan, α-(1,3)-glucan, and chitin, which together constitute nearly 80% of cell wall polysaccharides (45). To dissect the mechanism of Wnt/β-catenin pathway activation by A. fumigatus swollen conidia, we analyzed the ability of various PAMPs of the A. fumigatus cell wall to induce the expression of active β-catenin and p-GSK-3β. Interestingly, only β-(1,3)-glucan and α-(1,3)-glucan, but not chitin, could activate the Wnt/β-catenin signaling (Fig. 7). Indeed, the level of expression of active β-catenin by β-(1,3)-glucan and α-(1,3)-glucan was on par with that of swollen conidia. Activation of the β-catenin pathway by these two glucans was in line with our previous report that both α-(1,3)-glucan and β-(1,3)-glucan induce DC-mediated polarization of Tregs from naive CD4^+^ T cells (37). However, the effects of the stimulation with swollen conidia on the phosphorylation of GSK-3β were higher than those promoted individually by β-(1,3)-glucan or α-(1,3)-glucan. This suggests that other polysaccharide PAMPs exposed on the swollen conidial surface might also play a role in the activation of the Wnt/β-catenin pathway in human DCs.

### Induction of the β-catenin pathway by A. fumigatus in DCs requires C-type lectin receptors.

**FIG 8:**
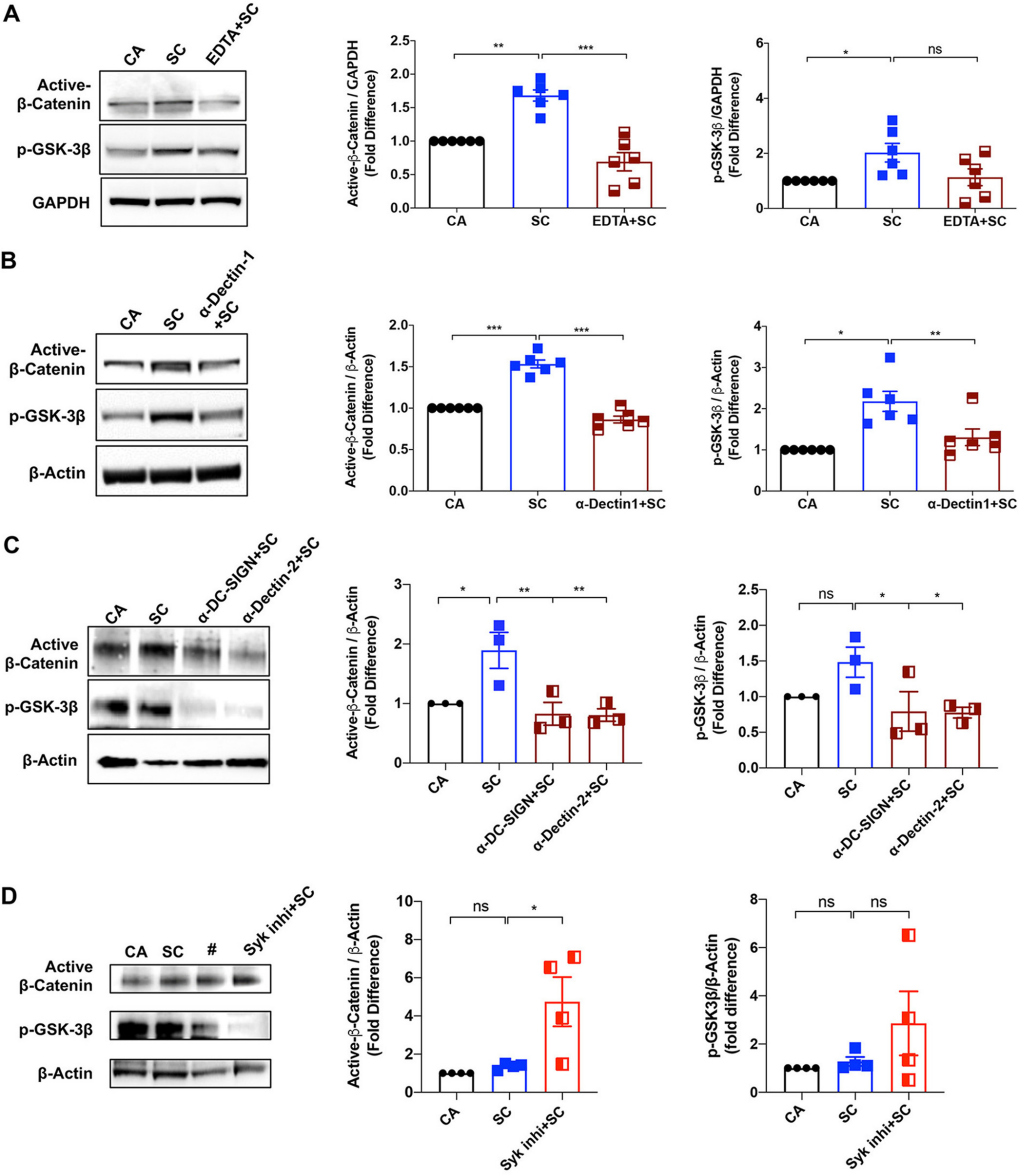
Induction of β-catenin pathway by A. fumigatus in DCs requires C-type lectin receptors. DCs were cultured with GM-CSF and IL-4 and were either left unstimulated (CA) or stimulated with swollen conidia (SC) or were treated with EDTA (A), or anti-Dectin-1 monoclonal antibody (B), or anti-DC-SIGN monoclonal antibody or anti-Dectin-2 monoclonal antibody (C), or Syk inhibitor (D) for 1 h before stimulating with swollen conidia. GAPDH (A) or β-actin (B to D) were used as loading controls. As EDTA chelates divalent cations such as Ca^+2^ and Mg^+2^, it could affect β-actin polymerization and stability. Hence, GAPDH was used as loading control for the experiment shown in panel A. Representative blots and densitometric analysis (mean ± SEM) of active β-catenin and p-GSK-3β from *n* = 6 (A and B), *n* = 3 (C) and *n* = 4 (D) experiments are presented. *, *P* < 0.05; **, *P* < 0.01; ***, *P* < 0.001; ns, not significant as determined by one-way ANOVA and Dunnett’s multiple-comparison test. The “#” symbol denotes irrelevant bands.

C-type lectin receptors like dectin-1, dectin-2, and dendritic cell-specific intercellular adhesion molecule 3 (ICAM3)-grabbing nonintegrin (DC-SIGN) are the main PRRs on DCs involved in the recognition of A. fumigatus, depending on the growth stage (12, 46–50). We investigated the broad implication of these receptors by chelating Ca^2+^ using EDTA before stimulation with swollen conidia, as these receptors require divalent metal ions for the recognition of fungal PAMPs. EDTA treatment drastically decreased the capacity of swollen conidia to induce the Wnt/β-catenin pathway in DCs, as evidenced by the reduced levels of active β-catenin and p-GSK-3β (Fig. 8A).

Next, we investigated the relative contribution of these C-type lectin receptors by using blocking monoclonal antibodies to dectin-1, dectin-2, or DC-SIGN. We found that individual blockade of these receptors on DCs led to a significant reduction in the expression of Wnt pathway intermediate proteins (Fig. 8B and C), thus confirming that interactions between various cell wall glucans of A. fumigatus and C-type lectin receptors play a predominant role in mediating the A. fumigatus-induced β-catenin pathway.

The signaling via C-type lectin receptors induces downstream molecules via Syk-dependent and Raf-1-dependent pathways (51, 52). Therefore, to investigate the relative importance of these pathways in the activation of the β-catenin signaling, we inhibited the Syk pathway before stimulation with swollen conidia. It is of note that the levels of active β-catenin were further enhanced upon inhibition of Syk (Fig. 8D), suggesting that Raf-1-dependent pathways might play a major role in the activation of the A. fumigatus-induced β-catenin pathway.

## DISCUSSION

Containment of pathogens and provoking effective immune response with minimal tissue damage are the critical functions of the host defense system. Being sentinels of the immune system, DCs play major roles in the recognition of pathogens, antigen processing and presentation, and polarizing T-cell responses. A plethora of PRRs in DCs recognize invading pathogens, and activation of ensuing various signaling cascades during this process dictates the nature of the immune response to a particular type of infection. Activation of the Wnt/β-catenin pathway during host-pathogen interaction is one of the mechanisms by which pathogens escape immune surveillance. Although this pathway is well studied in the case of bacterial infections, relatively little is known about its involvement during fungal pathogenesis (27–29).

Here, we show that stimulation of human DCs with swollen conidia of A. fumigatus leads to the activation of the Wnt/β-catenin pathway, along with the secretion of Wnt ligands Wnt1 and Wnt7a. On the other hand, dormant conidia failed to activate the β-catenin pathway, emphasizing the importance of conidial swelling (initiation of germination process) and exposure of cell wall components, particularly the polysaccharides to be recognized by PRRs on the DCs, to activate Wnt signaling. These data also indicate that due to the masking of polysaccharides and other PAMPs by the rodlet/melanin layer (34), dormant conidia are not able to activate innate immune cells and hence lack the capacity to induce the β-catenin pathway. Consistent with results of previous studies in mouse peritoneal macrophages (33) and THP-1 cells (32), we observed increased expression of *WNT5A* and *WNT3A* in human DCs upon stimulation with swollen conidia. However, we did not observe the secretion of these ligands in the cell-free supernatants, which might be because of unstable mRNA or because of incorrect posttranslational modifications by the acyltransferase Porcupine (PORCN), which is important for the secretion and function of Wnt proteins (53, 54).

The Wnt/β-catenin pathway alone or in cooperation with other signaling pathways plays an important role in the differentiation (55), maturation, and functions of DCs (56). The presence of Wnt ligands can modulate the DC response to various Toll-like receptor (TLR) stimuli by restraining the production of proinflammatory cytokines such as IL-6, IL-12, and TNF-α (20, 23). Stimulation of mouse DCs with Wnt ligands Wnt3a or Wnt5a makes them tolerogenic with the generation of anti-inflammatory cytokine (20). In general, Wnt condition DCs to a regulatory state in which they express higher levels of anti-inflammatory molecules like IL-10, TGF-β, retinoic acid, IL-27, and vascular endothelial growth factor (20–23). Hence, to understand the role of the active Wnt pathway in DC response to A. fumigatus, we employed Wnt inhibitor C-59, which blocks Porcupine-mediated Wnt palmitoylation and thereby inhibits the secretion and function of Wnt ligands. Inhibition of the Wnt/β-catenin pathway abrogated the swollen conidia-mediated maturation of DCs, as seen by decreased expression of DC surface markers such as CD83, CD80, CD86, CD40, and HLA-DR. However, Wnt inhibition did not affect the secretion of proinflammatory cytokines like IL-6, IL-8, and IL-12. These data are distinct from those obtained with mouse DCs, where stimulation of DCs with TLR9 ligand CpG in the presence of Wnt3a or Wnt5a did not affect the maturation of DCs (20). Also, Wnt5a, but not Wnt3a, could inhibit poly(I·C)-induced IL-6 production in these DCs (20). However, IL-12p40 was not altered by either of these ligands. Based on our data, we could conclude that the effect of Wnt ligands on either the maturation process of DCs or cytokine production depends on the PRR that senses the signal and/or on the Wnt ligands secreted. Thus, swollen conidia of A. fumigatus signaled human DCs mainly via C-type lectin receptors, and unlike in other reports, induced the secretion of Wnt1 and Wnt7a. These results might explain why blockade of the Wnt pathway in DCs prior to stimulation with swollen conidia led to the inhibition of maturation of DCs, while the production of inflammatory cytokines IL-6, IL-8, and IL-12 was not affected. It is of note that IL-10 induction appears to be a common feature of various Wnt ligands that activate the β-catenin pathway (20–23). Further fundamental studies are needed to dissect the role of individual PRRs in inducing distinct Wnt ligands and the effects of various Wnt ligands, particularly Wnt1 and Wnt7a, on the activation and functions of human DCs.

The dormant conidia of A. fumigatus are covered by the hydrophobin rodlet layer that masks the underlying cell wall polysaccharides. During germination of conidia, the outer rodlet layer is degraded, allowing the recognition of conidia by host PRRs (34). Major immunostimulatory polysaccharides of A. fumigatus exposed during germination process are the cell wall β-(1,3)-glucan, α-(1,3)-glucan, and chitin (35). In line with the fact that both β-(1,3)-glucan and α-(1,3)-glucan could induce Treg polarization, both of these polysaccharides activated the β-catenin pathway. These structural cell wall molecules are present in the cell walls of both swollen conidia and hyphae (6), which might explain the activation of the β-catenin pathway by both of these morphological forms of A. fumigatus. However, cell wall polysaccharides exhibit selectivity in their capacity to activate the β-catenin pathway, as chitin, for which FcγRII and intracellular PRRs are said to be the major receptors, failed to activate this pathway (57, 58).

Our data demonstrate that all major C-type lectin receptors—dectin-1, dectin-2, and DC-SIGN—that were reported to be implicated in the crosstalk between A. fumigatus and DCs (12, 46–50) have a role in mediating β-catenin activation. Dectin-1 and DC-SIGN induce Syk and Raf-1 signaling pathways in DCs that converge at the point of NF-κB activation (52). Further, engagement of the dectin-1 receptor by fungal pathogens is known to stabilize β-catenin via the Syk-ROS axis (33). Our data, however, suggest that the Raf-1 signaling pathway, but not Syk, has a major role in the activation of β-catenin. Additionally, dectin-1-mediated phosphorylation of Akt has been shown to activate the β-catenin pathway (59), either directly by phosphorylating β-catenin at Ser552 (60–62) or indirectly by preventing β-catenin degradation by phosphorylating GSK-3β at Ser9 (63). Likewise, C-type lectin receptors cooperate with TLR, for example, in the synergy between dectin-1 and TLR2. Therefore, PI3K-Akt pathways might also contribute to the A. fumigatus-induced activation of β-catenin (20, 23). The cell wall composition and localization of polysaccharides are dynamic processes and hence implication of a particular PRR would be also influenced by the growth stage of A. fumigatus.

The status of the Wnt/β-catenin pathway in DCs also influences the polarization of naive T cells. Activation of β-catenin in DCs in the intestine, either naturally by mucin or by experimental approaches, leads to enhanced generation of Tregs (64, 65). In contrast, lack of β-catenin signaling in DCs instigates exacerbation of diseases like colitis and experimental autoimmune encephalitis (EAE) because of the increased levels of Th1 and Th17 cells, which play critical roles in the disease progression (21, 23, 64). In line with these findings, we observed downregulation of Treg responses upon inhibition of the Wnt/β-catenin pathway in swollen conidia-stimulated DCs, while the frequency of IFN-γ-producing Th1 cells remained unaltered. Although, Tregs are important at later stages of the infection to control infection-mediated tissue damage, controlling these cells during the initial stages of acute infection is equally important to promote protective Th1 responses to Aspergillus (14, 66). Therefore, targeting the Wnt/β-catenin pathway could be a useful strategy to selectively suppress the Treg response and to sustain the protective Th1 response against A. fumigatus in invasive fungal diseases. Although the frequency of Th17 cells is higher in Aspergillus-infected lungs (67), we observed only a marginal increase in the IL-17^+^ CD4^+^ T cells. Our results are in line with the observation that A. fumigatus induces primarily a Th1 rather than Th17 response (14).

Mechanistically, the Wnt pathway controls the polarization of Tregs by inducing PD-L1 on DCs. A recent report, however, shows that PD-1 in Tregs exerts cell-intrinsic inhibitory effects by regulating the PI3-Akt signaling and metabolism of cells (68). While potent suppression by Tregs is important for immune tolerance, in the context of infection, it causes persistent and chronic infection due to the excessive suppression of effector T cells. A large number of studies in diverse models have also shown that irrespective of PD-1 expression, Tregs are immunosuppressive. Therefore, PD-1 possibly ensures a fine balance between protective T-cell response and suppression of excessive inflammation by Tregs that is critical for the clearance of the pathogens and prevention of inflammation-induced tissue damage (69).

The A. fumigatus cell wall polysaccharides α-(1,3)-glucan and β-(1,3)-glucan induce the expression of PD-L1 in DCs and promote Treg polarization (37). Several reports have demonstrated that the Wnt/β-catenin pathway controls the expression of PD-L1 in different tumor settings (70–72). In compliance with these findings, we observed a significant decrease in the expression of PD-L1 upon inhibition of the Wnt/β-catenin pathway. In addition to limiting the Treg response, the decreased PD-L1 levels upon Wnt/β-catenin inhibition would also reduce the T-cell exhaustion observed during severe fungal infection (73–76).

## MATERIALS AND METHODS

### Reagents and antibodies.

For the immunoblot analysis, following antibodies from Cell Signaling Technology (Ozyme, Saint Quentin Yvelines, France) were used: rabbit monoclonal antibodies (MAbs) to nonphospho (active) β-catenin (Ser33/37/Thr41) (clone D13A1), p-GSK-3β (Ser9) (clone D3A4), GAPDH (horseradish peroxidase conjugated [HRP], clone 14C10), β-actin (HRP conjugated, clone 13E5), and polyclonal antibodies to phospho (inactive) β-catenin (Ser33/37/Thr41).

For flow cytometry, the following fluorochrome-conjugated monoclonal antibodies were used: from BD Biosciences, HLA-DR-allophycocyanin (APC) (clone G46-6), CD8-PB (clone RPA-T8), IL-10-phycoerythrin (PE) (clone JES5-16E3), CD86-fluorescein isothiocyanate (FITC) (clone FUN-1), CD80-PE (clone L307.4), CD83-APC (clone HB15e), CD274-FITC (clone MIH1), CD252-PE (clone iK-1), CD275-PE (clone 2D3/B7-H2), CD273-PE (clone, MIH18), CD25-FITC (clone M-A251), CD127-BV421 (clone HIL-7RM21), CD279-APC (clone M1H4), IFN-γ-FITC (clone 4S.B3), IL-4-PE (clone MP4-25D2); from eBioscience: FoxP3-APC (clone 236A/E7), IL-2-APC (clone MQ1-17H12), IL-17A-PE (clone eBio64cap17), IL-17A-FITC (clone eBio64DEC1); from Beckman Coulter: CD40-PE (clone MAB89); from BioLegend: CD4-PerCP (clone SK3); and from Miltenyi Biotec, TNF-α-APC (clone cA2).

Cell viability was detected using the fixable viability dye eFluor 506 (eBioscience).

### Culture and isolation of A. fumigatus conidia.

An Aspergillus fumigatus clinical isolate obtained from an invasive aspergillosis patient (CBS144-89) (77) was the strain used in this study and was maintained on 2% malt agar slant at 23 to 25°C. A. fumigatus conidia (asexual spores) were harvested from 10- to 12-day-old slants using 0.05% aqueous Tween 80, filtered through a 40-μm Falcon cell strainer (Thermo Fisher Scientific) to remove any mycelial contamination, washed (2×), and resuspended in aqueous Tween 80. These dormant conidia were either used for DC stimulation or inoculated in Sabouraud liquid medium (1 × 10^8^ spores per 50 mL medium) and incubated at 37°C for 5 h (swollen conidia) or 7.5 to 8 h (for germinating/hyphae morphotype) in a shaken incubator maintained with 150 rpm. Next, swollen and germinating conidia were harvested and washed with water. All of the conidial morphotypes (dormant, swollen, and germinating/hyphae) were inactivated by fixing with paraformaldehyde (PFA) as described previously (34).

### Isolation of α-(1,3)-glucan, β-(1,3)-glucan, and chitin from A. fumigatus.

β-(1,3)-Glucan, α-(1,3)-glucan, and chitin were isolated from the A. fumigatus cell wall as we described before (37, 57, 78, 79). In brief, alkali-insoluble (AI) and alkali-soluble (AS) fractions were extracted from the cell wall of mycelial culture grown in Sabouraud liquid medium for 24 h at 37°C in a shaken incubator at 150 rpm. Following, AI and AS fractions were subjected to periodate oxidation and Smith degradation, which resulted in β-(1,3)-glucan linked to chitin and α-1,3-glucan in the AI and AS fractions, respectively. The residual β-(1,3)-glucan in the α-(1,3)-glucan preparation was removed by a recombinant endo-β-1,3-glucanase treatment. β-1,3-Glucan-linked chitin was destroyed by deacetylation and nitrous deamination. Chitin was extracted from the mycelial cell wall upon alkali extraction and oxidized with H_2_O_2_ at 121°C for 20 min (autoclave) in the presence of glacial acetic acid, followed by a second alkali extraction and then washing with water (57).

### Generation and culture of DCs.

The buffy coats of the healthy donors (Centre Necker-Cabanel, L'Établissement Français du Sang, Paris) (EFS-INSERM ethical committee permissions 15/EFS/012 and 18/EFS/033) were used to isolate peripheral blood mononuclear cells (PBMCs) by Ficoll density gradient centrifugation. Monocytes were positively selected from PBMCs using CD14 MicroBeads (Miltenyi Biotec, Paris, France) followed by culture with granulocyte-macrophage colony-stimulating factor (GM-CSF) (1,000 IU/10^6^ cells) (Miltenyi Biotec) and IL-4 (500 IU/10^6^ cells) (Miltenyi Biotec) in RPMI 1640 complemented with 10% fetal calf serum (FCS) for 5 days to obtain immature DCs.

Immature DCs (0.5 × 10^6^/mL) were cultured with GM-CSF and IL-4 and were either left unstimulated (cells alone) or stimulated with swollen conidia (0.5 × 10^6^/mL), dormant conidia (0.5 × 10^6^/mL), germinating/hyphae morphotype (0.5 × 10^6^/mL), live conidia (500/mL) or treated with α-(1,3)-glucan (1 μg/mL/0.5 × 10^6^ DCs), β-(1,3)-glucan (1 μg/mL/0.5 × 10^6^ DCs), or chitin (1 μg/mL/0.5 × 10^6^ DCs) as mentioned in the respective figure legends.

For the inhibition of the Wnt/β-catenin pathway, DCs were preincubated with pharmacological inhibitor C-59 (15 μM; Calbiochem, Merck Chimie SAS, Fontenay sous Bois, France) or vehicle control dimethyl sulfoxide (DMSO; Sigma-Aldrich) for 2 h, followed by stimulation with swollen conidia for 48 h. After the incubation period, cells were processed for surface staining for various markers and acquired using an LSR II instrument (BD Biosciences), and the data were analyzed by BD FACS DIVA and FlowJo software. Cell-free supernatants were saved for the various cytokine analyses.

In other experiments, DCs were treated with the chelating agent EDTA (0.5 mM; Invitrogen, Thermo Fisher Scientific, Illkirch, France), anti-dectin-1 monoclonal antibody (GE2; 8 μg/0.5 × 10^6^ DCs/mL) (50, 80), anti-dectin-2 monoclonal antibody (10 μg/0.5 × 10^6^ DCs/mL, InvivoGen), anti-DC-SIGN monoclonal antibody (10 μg/0.5 × 10^6^ DCs/mL, R&D Systems) (37), Syk inhibitor (5 μM, InvivoGen) (81) for 1 h, or with LiCl to inhibit GSK-3β (10 mM, Sigma-Aldrich) for 2 h, followed by stimulation with swollen conidia.

### Coculture of DCs and naive CD4^+^ T cells.

After treatment of DCs as mentioned in the above section, they were subjected to mixed lymphocyte reaction with autologous naive CD4^+^ CD45RA^+^ CD25^−^ T cells at a 1:10 DC to T cell ratio for 5 days in serum-free X-Vivo medium. Naive T cells were isolated from PBMCs using multiple selection processes. namely a CD4^+^ T-cell isolation kit to isolate CD4^+^ T cells and depletion of memory T cells using CD45RO microbeads followed by depletion of CD25^+^ cells using CD25 microbeads (all from Miltenyi Biotec). After 5 days of culture, cells were washed and activated with phorbol 12-myristate 13-acetate (50 ng/mL/0.5 × 10^6^ cells; Sigma-Aldrich) and ionomycin (500 ng/mL/0.5 × 10^6^ cells; Sigma-Aldrich) along with GolgiStop (BD Biosciences) for 4 h for analysis of T-cell polarization. Cells were stained for surface molecules (CD4, CD25, and CD127), followed by fixation and permeabilization using the FoxP3 fixation/permeabilization kit (eBioscience) and intracellular staining (IFN-γ, IL-4, IL-17A, IL-10, and FoxP3). The gating strategy for Tregs is presented in Fig. S4 in the supplemental material.

10.1128/mBio.02824-21.4 FIG S4 Gating strategy for Tregs. Download FIG S4, EPS file, 0.8 MB. Copyright © 2021 Karnam et al. 2021 Karnam et al. https://creativecommons.org/licenses/by/4.0/ This content is distributed under the terms of the Creative Commons Attribution 4.0 International license.

To investigate the role of PD-L1 on DCs in mediating the CD4^+^ T-cell responses, DCs were stimulated with swollen conidia for 48 h. Then, DCs were washed and incubated with anti-PD-L1 (10 μg/0.5 × 10^6^ DCs/mL, clone M1H1; Invitrogen) or isotype control monoclonal antibody (mouse IgG1 kappa, clone P3.6.2.8.1; Invitrogen) for 2 h (37) and then cocultured with naive CD4^+^ T cells.

### Coculture of DCs and CD8^+^ T cells.

DCs were cocultured with autologous CD8^+^ T cells at a 1:10 DC to T cell ratio for 5 days in serum-free X-Vivo medium. CD8^+^ T cells were isolated from PBMCs using CD8 micro beads (Miltenyi Biotec). After 5 days, cells were washed and activated with phorbol 12-myristate 13-acetate and ionomycin, along with GolgiStop, for 4 h. Cells were stained for the surface molecule (CD8), followed by intracellular staining (IFN-γ, IL-2, IL-10, and TNF-α).

### RNA isolation and quantitative RT-PCR.

For the Wnt gene expression analyses, cells were stimulated with swollen conidia or dormant conidia for 18 h. Unstimulated cells were used as a control. Total RNA from the different conditions was isolated using the RNeasy minikit (Qiagen, Hilden, Germany). cDNA was synthesized using a high-capacity cDNA reverse transcription kit (Thermo Fisher Scientific, Courtaboeuf, France). For quantitative reverse transcription-PCR (RT-PCR), SYBR green PCR master mix (Thermo Fisher Scientific) was used. Gene expression was calculated relative to the reference gene GAPDH.

The primers used in the study are as follows: WNT1-F, CAGCGACAACATTGACTTCG, and WNT1-R, GCCTCGTTGTTGTGAAGGTT; WNT3-F, GGAGAAGCGGAAGGAAAAATG, and WNT3-R, GCACGTCGTAGATGCGAATACA; WNT3A-F, CCTGCACTCCATCCAGCTACA, and WNT3A-R, GACCTCTCTTCCTACCTTTCCCTTA; WNT5A-F, CTCACTGAAATGCGTGTTGG, and WNT5A-R, AATGCCCTCTCCACAAAGTG; WNT7A-F, CCCACCTTCCTGAAGATCAA, and WNT7A-R, GTCCTCCTCGCAGTAGTTGG; WNT7B-F, GGCACAAGGACCTACCAGAG, and WNT7B-R, CCTGATGTGTTCTCCCAGGT; WNT8A-F, TGGGGAACCTGTTTATGCTC, and WNT8A-R, AGATAGGCCTTGGGACCTGT; and GAPDH-F, CGACCACTTTGTCAAGCTCA, and GAPDH-R, GGTGGTCCAGGGGTCTTACT.

### Immunoblotting.

For the immunoblot analysis of active β-catenin and p-GSK-3β (82), cells were lysed to obtain total cell proteins using radioimmunoprecipitation assay (RIPA) buffer (Sigma-Aldrich) with protease and phosphatase inhibitors (Sigma-Aldrich). Proteins were quantified using Bio-Rad protein assay reagent (Bio-Rad, Marnes-la-Coquette, France) and equal amounts of proteins from each sample were resolved on SDS-PAGE gels, followed by transfer to the nitrocellulose iBlot transfer stack (Thermo Fisher Scientific). After transfer, the membrane was blocked using 5% bovine serum albumin (BSA) in TBST (Tris-buffered saline [TBS] and polysorbate 20 or Tween 20) for 60 min, followed by incubation with various primary antibodies at 4°C overnight. Blots were then washed three times in TBST with 10-min intervals and probed with secondary antibody for 2 h. Blots were again washed three times in TBST and developed with SuperSignal West Dura extended duration substrate (Thermo Fisher Scientific). β-Actin or GAPDH were used as the loading controls. Western blot images were obtained from the iBright CL1000 imaging system and analyzed by iBright Analysis software (Thermo Fisher Scientific). The full Western blot images are provided in Fig. S5.

10.1128/mBio.02824-21.5 FIG S5 Full Western blot images. The Western blot images that are shown in the article are marked by boxes. The “#” symbol indicates an irrelevant band. Download FIG S5, EPS file, 2.8 MB. Copyright © 2021 Karnam et al. 2021 Karnam et al. https://creativecommons.org/licenses/by/4.0/ This content is distributed under the terms of the Creative Commons Attribution 4.0 International license.

### Enzyme-limited immunosorbent assay.

Cell free supernatants of DCs were analyzed for the secretion of cytokines IL-6, IL-8, IL-10, IL-12, IL-1β, and TNF-α (enzyme-limited immunosorbent assay [ELISA] Ready-SET-Go; eBioscience). Secretion of Wnt1, Wnt3a, Wnt5a, Wnt7a, and Wnt7b was analyzed in cell-free supernatants from DCs with the help of human Wingless type mouse mammary tumor virus (MMTV) integration site family, member 1 (WNT1) ELISA kit, human protein Wnt-3a, Wnt-5a ELISA kit, human protein Wnt-7a ELISA kit or human protein Wnt-7b ELISA kit (MyBioSource, San Diego, CA).

### Statistical analysis.

As highlighted in the figure legends, the experiments were repeated several times using cells from independent donors. Graphs and statistical analyses were performed by one-way analysis of variance (ANOVA) with Tukey’s or Dunnett’s multiple-comparison tests, as indicated, using Prism 8 (GraphPad Software, Inc., CA). A *P* value of <0.05 was considered significant.

## References

[1] Tillie-Leblond I, Tonnel A-B. 2005. Allergic bronchopulmonary aspergillosis. Allergy 60:1004–1013. doi:10.1111/j.1398-9995.2005.00887.x. 15969680

[2] Cockrill BAMD, Hales CAMD. 1999. Allergic bronchopulmonary aspergillosis. Annu Rev Med 50:303–316. doi:10.1146/annurev.med.50.1.303. 10073280

[3] Chakrabarti A, Kaur H. 2016. Allergic *Aspergillus* rhinosinusitis. J Fungi (Basel) 2:32. doi:10.3390/jof2040032. PMC5715928 29376948

[4] Pappas PG, Alexander BD, Andes DR, Hadley S, Kauffman CA, Freifeld A, Anaissie EJ, Brumble LM, Herwaldt L, Ito J, Kontoyiannis DP, Lyon GM, Marr KA, Morrison VA, Park BJ, Patterson TF, Perl TM, Oster RA, Schuster MG, Walker R, Walsh TJ, Wannemuehler KA, Chiller TM. 2010. Invasive fungal infections among organ transplant recipients: results of the Transplant-Associated Infection Surveillance Network (TRANSNET). Clin Infect Dis 50:1101–1111. doi:10.1086/651262. 20218876

[5] Perfect JR. 2012. The impact of the host on fungal infections. Am J Med 125:S39–51. doi:10.1016/j.amjmed.2011.10.010. 22196208

[6] van de Veerdonk FL, Gresnigt MS, Romani L, Netea MG, Latgé J-P. 2017. *Aspergillus fumigatus* morphology and dynamic host interactions. Nat Rev Microbiol 15:661–674. doi:10.1038/nrmicro.2017.90. 28919635

[7] Bozza S, Gaziano R, Spreca A, Bacci A, Montagnoli C, di Francesco P, Romani L. 2002. Dendritic cells transport conidia and hyphae of *Aspergillus fumigatus* from the airways to the draining lymph nodes and initiate disparate Th responses to the fungus. J Immunol 168:1362–1371. doi:10.4049/jimmunol.168.3.1362. 11801677

[8] Gafa V, Lande R, Gagliardi MC, Severa M, Giacomini E, Remoli ME, Nisini R, Ramoni C, Di Francesco P, Aldebert D, Grillot R, Coccia EM. 2006. Human dendritic cells following *Aspergillus fumigatus* infection express the CCR7 receptor and a differential pattern of interleukin-12 (IL-12), IL-23, and IL-27 cytokines, which lead to a Th1 response. Infect Immun 74:1480–1489. doi:10.1128/IAI.74.3.1480-1489.2006. 16495518 PMC1418673

[9] Ramirez-Ortiz ZG, Lee CK, Wang JP, Boon L, Specht CA, Levitz SM. 2011. A nonredundant role for plasmacytoid dendritic cells in host defense against the human fungal pathogen *Aspergillus fumigatus*. Cell Host Microbe 9:415–424. doi:10.1016/j.chom.2011.04.007. 21575912 PMC3100664

[10] Chamilos G, Ganguly D, Lande R, Gregorio J, Meller S, Goldman WE, Gilliet M, Kontoyiannis DP. 2010. Generation of IL-23 producing dendritic cells (DCs) by airborne fungi regulates fungal pathogenicity via the induction of T_H_-17 responses. PLoS One 5:e12955. doi:10.1371/journal.pone.0012955. 20886035 PMC2944889

[11] Mezger M, Kneitz S, Wozniok I, Kurzai O, Einsele H, Loeffler J. 2008. Proinflammatory response of immature human dendritic cells is mediated by dectin-1 after exposure to *Aspergillus fumigatus* germ tubes. J Infect Dis 197:924–931. doi:10.1086/528694. 18279049

[12] Serrano-Gómez D, Domínguez-Soto A, Ancochea J, Jimenez-Heffernan JA, Leal JA, Corbí AL. 2004. Dendritic cell-specific intercellular adhesion molecule 3-grabbing nonintegrin mediates binding and internalization of *Aspergillus fumigatus* conidia by dendritic cells and macrophages. J Immunol 173:5635–5643. doi:10.4049/jimmunol.173.9.5635. 15494514

[13] Loures FV, Röhm M, Lee CK, Santos E, Wang JP, Specht CA, Calich VLG, Urban CF, Levitz SM. 2015. Recognition of *Aspergillus fumigatus* hyphae by human plasmacytoid dendritic cells is mediated by dectin-2 and results in formation of extracellular traps. PLoS Pathog 11:e1004643. doi:10.1371/journal.ppat.1004643. 25659141 PMC4450068

[14] Chai LYA, Veerdonk FVD, Marijnissen RJ, Cheng S-C, Khoo AL, Hectors M, Lagrou K, Vonk AG, Maertens J, Joosten LAB, Kullberg B-J, Netea MG. 2010. Anti-*Aspergillus* human host defence relies on type 1 T helper (Th1), rather than type 17 T helper (Th17), cellular immunity. Immunology 130:46–54. doi:10.1111/j.1365-2567.2009.03211.x. 20002791 PMC2855792

[15] Cenci E, Mencacci A, Del Sero G, Bacci A, Montagnoli C, d’Ostiani CF, Mosci P, Bachmann M, Bistoni F, Kopf M, Romani L. 1999. Interleukin-4 causes susceptibility to invasive pulmonary aspergillosis through suppression of protective type I responses. J Infect Dis 180:1957–1968. doi:10.1086/315142. 10558953

[16] Centeno-Lima S, Silveira H, Casimiro C, Aguiar P, do Rosário VE. 2002. Kinetics of cytokine expression in mice with invasive aspergillosis: lethal infection and protection. FEMS Immunol Med Microbiol 32:167–173. doi:10.1111/j.1574-695X.2002.tb00549.x. 11821239

[17] Bozza S, Iannitti RG, Pariano M, Renga G, Costantini C, Romani L, Bayry J. 2021. Small molecule CCR4 antagonists protect mice from *Aspergillus* infection and allergy. Biomolecules 11:351. doi:10.3390/biom11030351. 33669094 PMC7996545

[18] Speakman EA, Dambuza IM, Salazar F, Brown GD. 2020. T cell antifungal immunity and the role of C-type lectin receptors. Trends Immunol 41:61–76. doi:10.1016/j.it.2019.11.007. 31813764 PMC7427322

[19] Montagnoli C, Fallarino F, Gaziano R, Bozza S, Bellocchio S, Zelante T, Kurup WP, Pitzurra L, Puccetti P, Romani L. 2006. Immunity and tolerance to *Aspergillus* involve functionally distinct regulatory T cells and tryptophan catabolism. J Immunol 176:1712–1723. doi:10.4049/jimmunol.176.3.1712. 16424201

[20] Oderup C, LaJevic M, Butcher EC. 2013. Canonical and noncanonical Wnt proteins program dendritic cell responses for tolerance. J Immunol 190:6126–6134. doi:10.4049/jimmunol.1203002. 23677472 PMC3698971

[21] Suryawanshi A, Manoharan I, Hong Y, Swafford D, Majumdar T, Taketo MM, Manicassamy B, Koni PA, Thangaraju M, Sun Z, Mellor AL, Munn DH, Manicassamy S. 2015. Canonical Wnt signaling in dendritic cells regulates Th1/Th17 responses and suppresses autoimmune neuroinflammation. J Immunol 194:3295–3304. doi:10.4049/jimmunol.1402691. 25710911 PMC4369436

[22] Valencia J, Hernández-López C, Martínez VG, Hidalgo L, Zapata AG, Vicente Á, Varas A, Sacedón R. 2011. Wnt5a skews dendritic cell differentiation to an unconventional phenotype with tolerogenic features. J Immunol 187:4129–4139. doi:10.4049/jimmunol.1101243. 21918189

[23] Manoharan I, Hong Y, Suryawanshi A, Angus-Hill ML, Sun Z, Mellor AL, Munn DH, Manicassamy S. 2014. TLR2-dependent activation of β-catenin pathway in dendritic cells induces regulatory responses and attenuates autoimmune inflammation. J Immunol 193:4203–4213. doi:10.4049/jimmunol.1400614. 25210120 PMC4185231

[24] Shen C-C, Kang Y-H, Zhao M, He Y, Cui D-D, Fu Y-Y, Yang L-L, Gou L-T. 2014. WNT16B from ovarian fibroblasts induces differentiation of regulatory T cells through β-catenin signal in dendritic cells. Int J Mol Sci 15:12928–12939. doi:10.3390/ijms150712928. 25050785 PMC4139882

[25] Clevers H, Nusse R. 2012. Wnt/β-catenin signaling and disease. Cell 149:1192–1205. doi:10.1016/j.cell.2012.05.012. 22682243

[26] Staal FJT, Luis TC, Tiemessen MM. 2008. WNT signalling in the immune system: WNT is spreading its wings. Nat Rev Immunol 8:581–593. doi:10.1038/nri2360. 18617885

[27] Ljungberg JK, Kling JC, Tran TT, Blumenthal A. 2019. Functions of the WNT signaling network in shaping host responses to infection. Front Immunol 10:2521. doi:10.3389/fimmu.2019.02521. 31781093 PMC6857519

[28] Jati S, Sarraf TR, Naskar D, Sen M. 2019. Wnt signaling: pathogen incursion and immune defense. Front Immunol 10:2551. doi:10.3389/fimmu.2019.02551. 31736969 PMC6828841

[29] Mukherjee T, Balaji KN. 2019. The WNT framework in shaping immune cell responses during bacterial infections. Front Immunol 10:1985. doi:10.3389/fimmu.2019.01985. 31497020 PMC6712069

[30] Silva-García O, Valdez-Alarcón JJ, Baizabal-Aguirre VM. 2014. The Wnt/β-catenin signaling pathway controls the inflammatory response in infections caused by pathogenic bacteria. Mediators Inflamm 2014:310183. doi:10.1155/2014/310183. 25136145 PMC4127235

[31] Spinnler K, Mezger M, Steffens M, Sennefelder H, Kurzai O, Einsele H, Loeffler J. 2010. Role of glycogen synthase kinase 3 (GSK-3) in innate immune response of human immature dendritic cells to *Aspergillus fumigatus*. Med Mycol 48:589–597. doi:10.3109/13693780903420625. 20055739

[32] Che C, Li C, Lin J, Zhang J, Jiang N, Yuan K, Zhao G. 2018. Wnt5a contributes to dectin-1 and LOX-1 induced host inflammatory response signature in *Aspergillus fumigatus* keratitis. Cell Signal 52:103–111. doi:10.1016/j.cellsig.2018.08.020. 30172652

[33] Trinath J, Holla S, Mahadik K, Prakhar P, Singh V, Balaji KN. 2014. The WNT signaling pathway contributes to dectin-1-dependent inhibition of Toll-like receptor-induced inflammatory signature. Mol Cell Biol 34:4301–4314. doi:10.1128/MCB.00641-14. 25246634 PMC4248746

[34] Aimanianda V, Bayry J, Bozza S, Kniemeyer O, Perruccio K, Elluru SR, Clavaud C, Paris S, Brakhage AA, Kaveri SV, Romani L, Latgé J-P. 2009. Surface hydrophobin prevents immune recognition of airborne fungal spores. Nature 460:1117–1121. doi:10.1038/nature08264. 19713928

[35] Valsecchi I, Stephen-Victor E, Wong SSW, Karnam A, Sunde M, Guijarro JI, Rodríguez de Francisco B, Krüger T, Kniemeyer O, Brown GD, Willment JA, Latgé J-P, Brakhage AA, Bayry J, Aimanianda V. 2020. The role of RodA-conserved cysteine residues in the *Aspergillus fumigatus* conidial surface organization. JoF 6:151. doi:10.3390/jof6030151. PMC7558875 32859091

[36] Nie X, Liu H, Liu L, Wang Y-D, Chen W-D. 2020. Emerging roles of Wnt ligands in human colorectal cancer. Front Oncol 10:1341. doi:10.3389/fonc.2020.01341. 32923386 PMC7456893

[37] Stephen-Victor E, Karnam A, Fontaine T, Beauvais A, Das M, Hegde P, Prakhar P, Holla S, Balaji KN, Kaveri SV, Latgé J-P, Aimanianda V, Bayry J. 2017. *Aspergillus fumigatus* cell wall α-(1,3)-glucan stimulates regulatory T-cell polarization by inducing PD-L1 expression on human dendritic cells. J Infect Dis 216:1281–1294. doi:10.1093/infdis/jix469. 28968869

[38] Potenza L, Vallerini D, Barozzi P, Riva G, Forghieri F, Beauvais A, Beau R, Candoni A, Maertens J, Rossi G, Morselli M, Zanetti E, Quadrelli C, Codeluppi M, Guaraldi G, Pagano L, Caira M, Giovane CD, Maccaferri M, Stefani A, Morandi U, Tazzioli G, Girardis M, Delia M, Specchia G, Longo G, Marasca R, Narni F, Merli F, Imovilli A, Apolone G, Carvalho A, Comoli P, Romani L, Latgè JP, Luppi M. 2013. Characterization of specific immune responses to different *Aspergillus* Antigens during the course of invasive aspergillosis in hematologic patients. PLoS One 8:e74326. doi:10.1371/journal.pone.0074326. 24023936 PMC3762751

[39] Bour-Jordan H, Esensten JH, Martinez‐Llordella M, Penaranda C, Stumpf M, Bluestone JA. 2011. Intrinsic and extrinsic control of peripheral T-cell tolerance by costimulatory molecules of the CD28/B7 family. Immunol Rev 241:180–205. doi:10.1111/j.1600-065X.2011.01011.x. 21488898 PMC3077803

[40] Francisco LM, Sage PT, Sharpe AH. 2010. The PD-1 pathway in tolerance and autoimmunity. Immunol Rev 236:219–242. doi:10.1111/j.1600-065X.2010.00923.x. 20636820 PMC2919275

[41] Simpson TR, Quezada SA, Allison JP. 2010. Regulation of CD4 T cell activation and effector function by inducible costimulator (ICOS). Curr Opin Immunol 22:326–332. doi:10.1016/j.coi.2010.01.001. 20116985

[42] Ruby CE, Yates MA, Hirschhorn-Cymerman D, Chlebeck P, Wolchok JD, Houghton AN, Offner H, Weinberg AD. 2009. Cutting Edge: OX40 agonists can drive regulatory T cell expansion if the cytokine milieu is right. J Immunol 183:4853–4857. doi:10.4049/jimmunol.0901112. 19786544 PMC4625917

[43] Sharpe AH, Wherry EJ, Ahmed R, Freeman GJ. 2007. The function of programmed cell death 1 and its ligands in regulating autoimmunity and infection. Nat Immunol 8:239–245. doi:10.1038/ni1443. 17304234

[44] Trinath J, Maddur MS, Kaveri SV, Balaji KN, Bayry J. 2012. Mycobacterium tuberculosis promotes regulatory T-cell expansion via induction of programmed death-1 ligand 1 (PD-L1, CD274) on dendritic cells. J Infect Dis 205:694–696. doi:10.1093/infdis/jir820. 22238465

[45] Kang X, Kirui A, Muszyński A, Widanage MCD, Chen A, Azadi P, Wang P, Mentink-Vigier F, Wang T. 2018. Molecular architecture of fungal cell walls revealed by solid-state NMR_supli. Nat Commun 9:2747. doi:10.1038/s41467-018-05199-0. 30013106 PMC6048167

[46] Cambi A, Gijzen K, de Vries IJM, Torensma R, Joosten B, Adema GJ, Netea MG, Kullberg B-J, Romani L, Figdor CG. 2003. The C-type lectin DC-SIGN (CD209) is an antigen-uptake receptor for *Candida albicans* on dendritic cells. Eur J Immunol 33:532–538. doi:10.1002/immu.200310029. 12645952

[47] Caparrós E, Munoz P, Sierra-Filardi E, Serrano-Gómez D, Puig-Kröger A, Rodríguez-Fernández JL, Mellado M, Sancho J, Zubiaur M, Corbí AL. 2006. DC-SIGN ligation on dendritic cells results in ERK and PI3K activation and modulates cytokine production. Blood 107:3950–3958. doi:10.1182/blood-2005-03-1252. 16434485

[48] Hardison SE, Brown GD. 2012. C-type lectin receptors orchestrate antifungal immunity. 9. Nat Immunol 13:817–822. doi:10.1038/ni.2369. 22910394 PMC3432564

[49] Taylor PR, Tsoni SV, Willment JA, Dennehy KM, Rosas M, Findon H, Haynes K, Steele C, Botto M, Gordon S, Brown GD. 2007. Dectin-1 is required for beta-glucan recognition and control of fungal infection. Nat Immunol 8:31–38. doi:10.1038/ni1408. 17159984 PMC1888731

[50] Steele C, Rapaka RR, Metz A, Pop SM, Williams DL, Gordon S, Kolls JK, Brown GD. 2005. The beta-glucan receptor dectin-1 recognizes specific morphologies of *Aspergillus fumigatus*. PLoS Pathog 1:e42. doi:10.1371/journal.ppat.0010042. 16344862 PMC1311140

[51] Tang J, Lin G, Langdon WY, Tao L, Zhang J. 2018. Regulation of C-type lectin receptor-mediated antifungal immunity. Front Immunol 9:123. doi:10.3389/fimmu.2018.00123. 29449845 PMC5799234

[52] Gringhuis SI, den Dunnen J, Litjens M, van der Vlist M, Wevers B, Bruijns SCM, Geijtenbeek TBH. 2009. Dectin-1 directs T helper cell differentiation by controlling noncanonical NF-κB activation through Raf-1 and Syk. Nat Immunol 10:203–213. doi:10.1038/ni.1692. 19122653

[53] Kadowaki T, Wilder E, Klingensmith J, Zachary K, Perrimon N. 1996. The segment polarity gene porcupine encodes a putative multitransmembrane protein involved in Wingless processing. Genes Dev 10:3116–3128. doi:10.1101/gad.10.24.3116. 8985181

[54] Hausmann G, Bänziger C, Basler K. 2007. Helping Wingless take flight: how WNT proteins are secreted. Nat Rev Mol Cell Biol 8:331–336. doi:10.1038/nrm2141. 17342185

[55] Zhou J, Cheng P, Youn J-I, Cotter MJ, Gabrilovich DI. 2009. Notch and Wingless signaling cooperate in regulation of dendritic cell differentiation. Immunity 30:845–859. doi:10.1016/j.immuni.2009.03.021. 19523851 PMC2700307

[56] Xu W, Wang J, Yuan T, Li Y, Yang H, Liu Y, Zhao Y, Herrmann M. 2016. Interactions between canonical Wnt signaling pathway and MAPK pathway regulate differentiation, maturation and function of dendritic cells. Cell Immunol 310:170–177. doi:10.1016/j.cellimm.2016.09.006. 27641635

[57] Becker KL, Aimanianda V, Wang X, Gresnigt MS, Ammerdorffer A, Jacobs CW, Gazendam RP, Joosten L.aB, Netea MG, Latgé JP, van de Veerdonk FL. 2016. *Aspergillus* cell wall chitin induces anti- and proinflammatory cytokines in human PBMCs via the Fc-γ receptor/Syk/PI3K pathway. mBio 7:e01823-15. doi:10.1128/mBio.01823-15. 27247234 PMC4895119

[58] Wagener J, Malireddi RKS, Lenardon MD, Köberle M, Vautier S, MacCallum DM, Biedermann T, Schaller M, Netea MG, Kanneganti T-D, Brown GD, Brown AJP, Gow NAR. 2014. Fungal chitin dampens inflammation through IL-10 induction mediated by NOD2 and TLR9 activation. PLoS Pathog 10:e1004050. doi:10.1371/journal.ppat.1004050. 24722226 PMC3983064

[59] Dennehy KM, Willment JA, Williams DL, Brown GD. 2009. Reciprocal regulation of IL-23 and IL-12 following co-activation of dectin-1 and TLR signaling pathways. Eur J Immunol 39:1379–1386. doi:10.1002/eji.200838543. 19291703 PMC2720084

[60] Fang D, Hawke D, Zheng Y, Xia Y, Meisenhelder J, Nika H, Mills GB, Kobayashi R, Hunter T, Lu Z. 2007. Phosphorylation of beta-catenin by AKT promotes beta-catenin transcriptional activity. J Biol Chem 282:11221–11229. doi:10.1074/jbc.M611871200. 17287208 PMC1850976

[61] Gantner BN, Jin H, Qian F, Hay N, He B, Ye RD. 2012. The Akt1 isoform is required for optimal IFN-β transcription through direct phosphorylation of β-catenin. J Immunol 189:3104–3111. doi:10.4049/jimmunol.1201669. 22904301 PMC3658160

[62] Yang P, An H, Liu X, Wen M, Zheng Y, Rui Y, Cao X. 2010. The cytosolic nucleic acid sensor LRRFIP1 mediates the production of type I interferon via a β-catenin-dependent pathway. Nat Immunol 11:487–494. doi:10.1038/ni.1876. 20453844

[63] Hay N. 2011. Interplay between FOXO, TOR, and Akt. Biochim Biophys Acta 1813:1965–1970. doi:10.1016/j.bbamcr.2011.03.013. 21440577 PMC3427795

[64] Manicassamy S, Reizis B, Ravindran R, Nakaya H, Salazar-Gonzalez RM, Wang Y, Pulendran B. 2010. Activation of β-Catenin in dendritic cells regulates immunity versus tolerance in the intestine. Science 329:849–853. doi:10.1126/science.1188510. 20705860 PMC3732486

[65] Shan M, Gentile M, Yeiser JR, Walland AC, Bornstein VU, Chen K, He B, Cassis L, Bigas A, Cols M, Comerma L, Huang B, Blander JM, Xiong H, Mayer L, Berin C, Augenlicht LH, Velcich A, Cerutti A. 2013. Mucus enhances gut homeostasis and oral tolerance by delivering immunoregulatory signals. Science 342:447–453. doi:10.1126/science.1237910. 24072822 PMC4005805

[66] Stuehler C, Khanna N, Bozza S, Zelante T, Moretti S, Kruhm M, Lurati S, Conrad B, Worschech E, Stevanović S, Krappmann S, Einsele H, Latgé J-P, Loeffler J, Romani L, Topp MS. 2011. Cross-protective TH1 immunity against *Aspergillus fumigatus* and *Candida albicans*. Blood 117:5881–5891. doi:10.1182/blood-2010-12-325084. 21441461

[67] Jolink H, de Boer R, Hombrink P, Jonkers RE, van Dissel JT, Falkenburg JHF, Heemskerk MHM. 2017. Pulmonary immune responses against *Aspergillus fumigatus* are characterized by high frequencies of IL-17 producing T-cells. J Infect 74:81–88. doi:10.1016/j.jinf.2016.10.010. 27838522

[68] Tan CL, Kuchroo JR, Sage PT, Liang D, Francisco LM, Buck J, Thaker YR, Zhang Q, McArdel SL, Juneja VR, Lee SJ, Lovitch SB, Lian C, Murphy GF, Blazar BR, Vignali DAA, Freeman GJ, Sharpe AH. 2020. PD-1 restraint of regulatory T cell suppressive activity is critical for immune tolerance. J Exp Med 218:e20182232. doi:10.1084/jem.20182232. PMC7543091 33045061

[69] Stephen-Victor E, Bosschem I, Haesebrouck F, Bayry J. 2017. The Yin and Yang of regulatory T cells in infectious diseases and avenues to target them. Cell Microbiol 19:e12746. doi:10.1111/cmi.12746. 28382773

[70] Castagnoli L, Cancila V, Cordoba-Romero SL, Faraci S, Talarico G, Belmonte B, Iorio MV, Milani M, Volpari T, Chiodoni C, Hidalgo-Miranda A, Tagliabue E, Tripodo C, Sangaletti S, Di Nicola M, Pupa SM. 2019. WNT signaling modulates PD-L1 expression in the stem cell compartment of triple-negative breast cancer. Oncogene 38:4047–4060. doi:10.1038/s41388-019-0700-2. 30705400 PMC6755989

[71] Du L, Lee J-H, Jiang H, Wang C, Wang S, Zheng Z, Shao F, Xu D, Xia Y, Li J, Zheng Y, Qian X, Li X, Kim H-R, Xing D, Liu P, Lu Z, Lyu J. 2020. β-Catenin induces transcriptional expression of PD-L1 to promote glioblastoma immune evasion. J Exp Med 217:e20191115. doi:10.1084/jem.20191115. 32860047 PMC7596815

[72] Li C-W, Lim S-O, Xia W, Lee H-H, Chan L-C, Kuo C-W, Khoo K-H, Chang S-S, Cha J-H, Kim T, Hsu JL, Wu Y, Hsu J-M, Yamaguchi H, Ding Q, Wang Y, Yao J, Lee C-C, Wu H-J, Sahin AA, Allison JP, Yu D, Hortobagyi GN, Hung M-C. 2016. Glycosylation and stabilization of programmed death ligand-1 suppresses T-cell activity. Nat Commun 7:12632. doi:10.1038/ncomms12632. 27572267 PMC5013604

[73] Lázár-Molnár E, Gácser A, Freeman GJ, Almo SC, Nathenson SG, Nosanchuk JD. 2008. The PD-1/PD-L costimulatory pathway critically affects host resistance to the pathogenic fungus Histoplasma capsulatum. Proc Natl Acad Sci USA 105:2658–2663. doi:10.1073/pnas.0711918105. 18268348 PMC2268192

[74] Spec A, Shindo Y, Burnham C-AD, Wilson S, Ablordeppey EA, Beiter ER, Chang K, Drewry AM, Hotchkiss RS. 2016. T cells from patients with *Candida* sepsis display a suppressive immunophenotype. Crit Care 20:15. doi:10.1186/s13054-016-1182-z. 26786705 PMC4719210

[75] Chang KC, Burnham C-A, Compton SM, Rasche DP, Mazuski RJ, McDonough JS, Unsinger J, Korman AJ, Green JM, Hotchkiss RS. 2013. Blockade of the negative co-stimulatory molecules PD-1 and CTLA-4 improves survival in primary and secondary fungal sepsis. Crit Care 17:R85. doi:10.1186/cc12711. 23663657 PMC3706819

[76] Hebart H, Bollinger C, Fisch P, Sarfati J, Meisner C, Baur M, Loeffler J, Monod M, Latgé J-P, Einsele H. 2002. Analysis of T-cell responses to *Aspergillus fumigatus* antigens in healthy individuals and patients with hematologic malignancies. Blood 100:4521–4528. doi:10.1182/blood-2002-01-0265. 12393638

[77] Thau N, Monod M, Crestani B, Rolland C, Tronchin G, Latgé JP, Paris S. 1994. rodletless mutants of *Aspergillus fumigatus*. Infect Immun 62:4380–4388. doi:10.1128/iai.62.10.4380-4388.1994. 7927699 PMC303120

[78] Muszkieta L, Aimanianda V, Mellado E, Gribaldo S, Alcàzar-Fuoli L, Szewczyk E, Prevost M-C, Latgé J-P. 2014. Deciphering the role of the chitin synthase families 1 and 2 in the *in vivo* and *in vitro* growth of *Aspergillus fumigatus* by multiple gene targeting deletion. Cell Microbiol 16:1784–1805. doi:10.1111/cmi.12326. 24946720

[79] Richie DL, Hartl L, Aimanianda V, Winters MS, Fuller KK, Miley MD, White S, McCarthy JW, Latgé J-P, Feldmesser M, Rhodes JC, Askew DS. 2009. A role for the unfolded protein response (UPR) in virulence and antifungal susceptibility in *Aspergillus fumigatus*. PLoS Pathog 5:e1000258. doi:10.1371/journal.ppat.1000258. 19132084 PMC2606855

[80] Willment JA, Marshall ASJ, Reid DM, Williams DL, Wong SYC, Gordon S, Brown GD. 2005. The human β-glucan receptor is widely expressed and functionally equivalent to murine dectin-1 on primary cells. Eur J Immunol 35:1539–1547. doi:10.1002/eji.200425725. 15816015

[81] Galeotti C, Stephen-Victor E, Karnam A, Das M, Gilardin L, Maddur MS, Wymann S, Vonarburg C, Chevailler A, Dimitrov JD, Benveniste O, Bruhns P, Kaveri SV, Bayry J. 2019. Intravenous immunoglobulin induces IL-4 in human basophils by signaling through surface-bound IgE. J Allergy Clin Immunol 144:524–535.e8. doi:10.1016/j.jaci.2018.10.064. 30529242

[82] Karnam A, Rambabu N, Das M, Bou-Jaoudeh M, Delignat S, Käsermann F, Lacroix-Desmazes S, Kaveri SV, Bayry J. 2020. Therapeutic normal IgG intravenous immunoglobulin activates Wnt-β-catenin pathway in dendritic cells. Commun Biol 3:96. doi:10.1038/s42003-020-0825-4. 32132640 PMC7055225

